# Inflammasomes in Intestinal Disease: Mechanisms of Activation and Therapeutic Strategies

**DOI:** 10.3390/ijms252313058

**Published:** 2024-12-04

**Authors:** Viviana Scalavino, Emanuele Piccinno, Gianluigi Giannelli, Grazia Serino

**Affiliations:** National Institute of Gastroenterology S. De Bellis, IRCCS Research Hospital, Via Turi 27, 70013 Castellana Grotte, BA, Italy; viviana.scalavino@irccsdebellis.it (V.S.); emanuele.piccinno@irccsdebellis.it (E.P.); gianluigi.giannelli@irccsdebellis.it (G.G.)

**Keywords:** inflammasome, gastrointestinal disease, miRNAs, inflammatory bowel disease, colorectal cancer

## Abstract

NOD-like receptors (NLRs) are a family of cytosolic pattern recognition receptors (PRRs) implicated in the innate immune sensing of pathogens and damage signals. NLRs act as sensors in multi-protein complexes called inflammasomes. Inflammasome activity is necessary for the maintenance of intestinal homeostasis, although their aberrant activation contributes to the pathogenesis of several gastrointestinal diseases. In this review, we summarize the main features of the predominant types of inflammasomes involved in gastrointestinal immune responses and their implications in intestinal disease, including Irritable Bowel Syndrome (IBS), Inflammatory Bowel Disease (IBD), celiac disease, and Colorectal Cancer (CRC). In addition, we report therapeutic discoveries that target the inflammasome pathway, highlighting promising novel therapeutic strategies in the treatment of intestinal diseases. Collectively, our understanding of the mechanisms of intestinal inflammasome activation and their interactions with other immune pathways appear to be not fully elucidated. Moreover, the clinical relevance of the efficacy of inflammasome inhibitors has not been evaluated. Despite these limitations, a greater understanding of the effectiveness, specificity, and reliability of pharmacological and natural inhibitors that target inflammasome components could be an opportunity to develop new therapeutic options for the treatment of intestinal disease.

## 1. Introduction

The innate immune system is the first line of the host’s defense against pathogens and endogenous danger signals. In the gastrointestinal tract, microorganisms colonize the mucosal surface and lumen and interact with the immune system to maintain the balance between immune activation and tolerance [1]. Pattern recognition receptors (PRRs) play a crucial role in the surveillance mechanisms of the innate immune system. They are proteins expressed by immune cells such as dendritic cells, macrophages, monocytes, neutrophils, and epithelial cells, and have the role of recognizing pathogen-associated molecular patterns (PAMPs) and damage-associated molecular patterns (DAMPs), as well as initiating the innate immune responses [2]. PRRs feature different classes depending on their cell localization and include transmembrane proteins such as Toll-like receptors (TLRs) and C-type lectin receptors (CLRs) and cytoplasmic proteins such as the Retinoic acid-inducible gene (RIG)-I-like receptors (RLRs) and NOD-like receptors (NLRs) [2]. NLRs constitute a family of sensor proteins which, once activated, provide a scaffold for the assembly of the multiprotein complex known as inflammasome. In the innate immune system, inflammasomes are cytosolic complexes that activate inflammatory responses and control the host’s defense against pathogens [3]. Inflammasomes regulate enzymes that can result in the release of pro-inflammatory cytokines and initiate a special form of programmed cell death known as pyroptosis [3]. According to recent evidence, inflammasome activity is crucial for intestinal homeostasis and plays an important role in discriminating pathogenic bacteria from commensal organisms [4]. Indeed, in recent years, several studies have reported a key role for inflammasomes in the pathogenesis of gastrointestinal diseases including celiac disease, irritable bowel syndrome (IBS), inflammatory bowel diseases (IBD) comprising Crohn’s Disease (CD) and Ulcerative Colitis (UC), and cancer [4].

In this review, we summarize the structures and functions of inflammasomes in the intestinal tract and their involvement in the pathogenesis of intestinal diseases. Furthermore, we discuss the potential therapeutic strategies targeting inflammasome pathways that are applicable in intestinal diseases. 

## 2. Inflammasome Sensors

The term “inflammasome” was coined by Martinon and colleagues around the turn of the 21st century to indicate a family of cytoplasmic proteins implicated in the activation of caspase-1 by the TLRs in activated immune cells [5]. Generally, in immune cells, inflammasomes are able to detect a broad range of endogenous or exogenous stimuli and induce cellular responses and effector release [6,7].

Inflammasome complexes are generally assigned to the categories of canonical and non-canonical inflammasomes. In canonical inflammasome assembly, sensors recognize ligands, triggering sensor oligomerization. Subsequently, the oligomers require an adaptor protein apoptosis-associated speck-like protein containing a caspase recruitment domain (ASC) that brings about effector pro-caspase-1 activation by proteolytic cleavage. Then, active caspase-1 triggers the cleavage and release of the active forms of interleukin (IL)-1β and IL-18 [8]. In addition, caspase-1 cleaves Gasdermin (GSDM) D which, by forming non-selective pores on the cell membrane, leads to the release of cytokines, cell swelling, and pyroptosis [9]. Non-canonical inflammasomes are a distinct type of inflammasome that are triggered by lipopolysaccharide (LPS) and act independently from caspase-1 by engaging the activation of caspase-4 and caspase-5 in humans and caspase-11 in mice. The caspases have the ability to function as sensors as well as effector molecules. In any case, the final outcomes are IL-1β and IL-18 release, activation of GSDMD, and pyroptosis [10].

**Figure 1:**
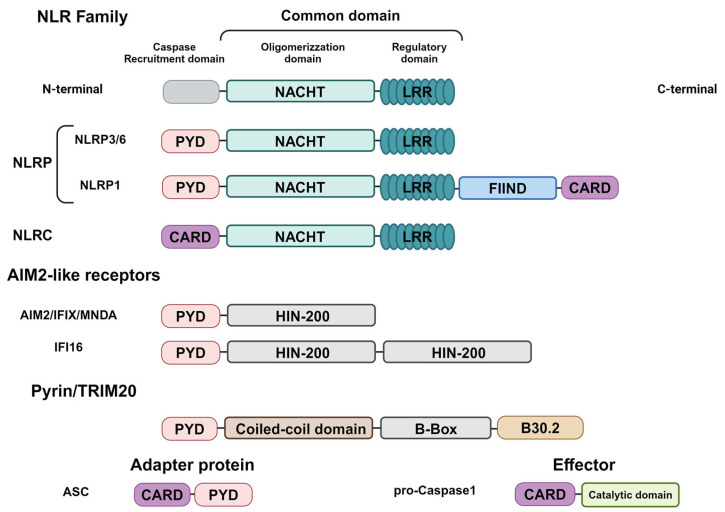
Domain structures of inflammasome components. NLR sensors contain a common central domain composed of a nucleotide-binding domain (NBD) and leucine-rich-repeat domain (LRR), but differ in regards to the N-Terminal domain required for Caspase-1 recruitment, consisting of the PYRIN domain (PYD) and caspase activation and recruitment domain (CARD). Moreover, the NLRP1 sensors differ in regard to the function-to-find domain (FIIND) and the CARD domain in the C-terminal. AIM2-like receptor sensors are composed of the N-Terminal PYD and C-Terminal IFN-inducible nuclear protein with 200-amino acids (HIN-200). Pyrin/TRIM20 sensors are composed of the N-Terminal PYD; a central region consists of an α-helical coiled-coil domain (C-C) and a zing finger domain (B-Box), as well as a C-terminal B30.2/SPRY domain. The adapter protein ASC is composed of a CARD and a PYD, whereas the effector protein pro-caspase-1 consists of a CARD domain and a catalytic domain. Created in BioRender.com.

The sensors’ molecules trigger the formation of inflammasomes following the recognition of inflammatory stimuli (Figure 1). The first group of sensors discovered, and now well described, contains a nucleotide oligomerization domain (NOD)-like receptor (NLR) sensor molecule [11]. They are the intracellular proteins that play a critical role in the regulation of the host’s innate immune response and include NLRP1, NLRP3, NLRP6, NLRP7, and NLRC4 [6,12]. Other proteins assembling canonical inflammasomes include the absent in melanoma 2 (AIM2)-like receptor (ALR), AIM2, and pyrin [12].

The NLR family comprises 23 genes in humans and 34 genes in mice [13]. The two most prominent subfamilies are the NLRC and NLRP groups of proteins [13]. Structurally, NLR proteins contain a central NACHT nucleotide-binding domain (NBD) and carboxy-terminal leucine-rich repeats (LRRs), but differ in terms of their N-terminal, since the NLRC proteins have one or more caspase recruitment (CARD) domains whereas the NLRP proteins have pyrin domains (PYD) at their N-terminal [13,14]. The NACHT domain has ATPase activity promoting the oligomerization of proteins, whereas the LRR domain has a regulatory function in promoting ligand binding. Furthermore, the N-terminal domain interacts with other proteins that carry out effector functions [13,14].

### 2.1. NLRP1

**Figure 2:**
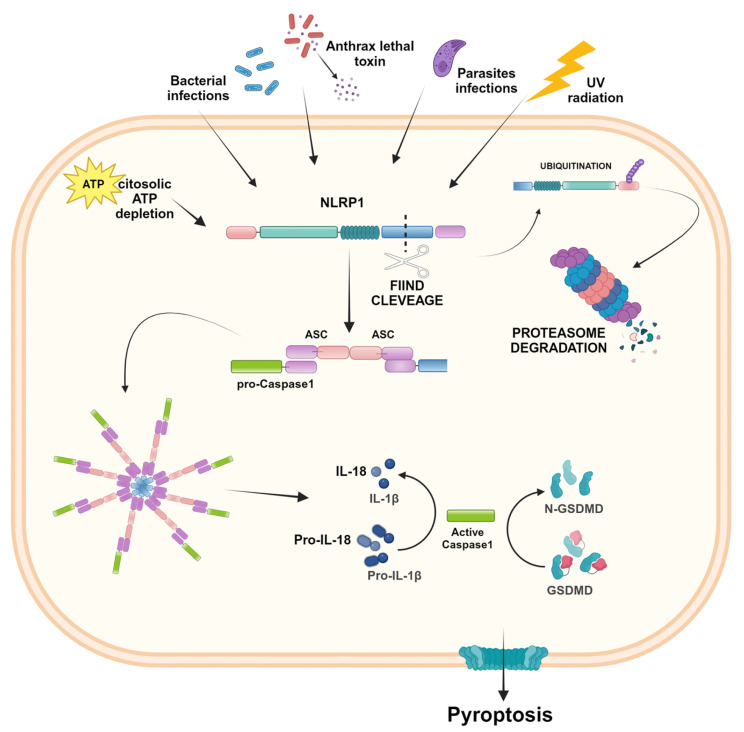
NLRP1 inflammasome activation. In the presence of bacterial and parasites infections, ATP depletion, and UV radiation, NLRP1 undergoes FIIND cleavage. The N-terminal domain is ubiquitinated and degraded by proteasomes, whereas the resulting FIIND-CARD fragment recruits the adapter protein ASC, which brings in pro-caspase-1, leading to the inflammasome complex. After proteolytic cleavage, the active caspase-1 results in IL-1β and IL-18 activation and the induction of pyroptosis. Created in BioRender.com.

NLRP1 was the first protein identified to carry out the activities of forming the inflammasome complex and activating caspase-1 [5] (Figure 2).

NLRP1 differs structurally from other NLR proteins because, in addition to the common domains, it contains a function-to-find domain (FIIND) and a C-terminal CARD. This domain critically requires the adaptor protein ASC for the recruitment of caspase-1 [12,15]. FIIND is constituted of two separated subdomains associated in a non-covalent manner and plays a crucial role in inhibiting the complete degradation of NLRP1 by the proteasomes. After inflammatory stimulation, the NLRP1 protein undergoes proteolysis of the FIIND level, and the N-terminal domain is ubiquitinated and degraded by the proteasome. After N-Terminal degradation, the subsequent FIIND-CARD fragment is released and acts as a platform for caspase-1 maturation and downstream signaling of inflammasomes [16]. In humans, a single NLRP1 gene is encoded, whereas in mice, paralogs have been found, namely NLRP1A, NLRP1B, and NLRP1C [12]. Mouse and human NLRP1 share common architectural features, except for PYD. The PYD necessary for recruiting ASC is present in human NLRP1, but is absent in murine NLRP1, which directly recruits caspase-1 through its CARD domain [17]. NLRP1 expression is widespread in different human organs and tissues, and several diseases are known to be associated with its dysfunction. In fact, NLRP1 was associated with autoimmune skin and lung disorders, arthritis, neurodegenerative disease, and cancer [17,18,19]. Moreover, polymorphisms in the NLRP1 gene were associated with CD, and aberrant activation of the NLRP1 inflammasome was involved in CRC [20].

### 2.2. NLRP3

**Figure 3:**
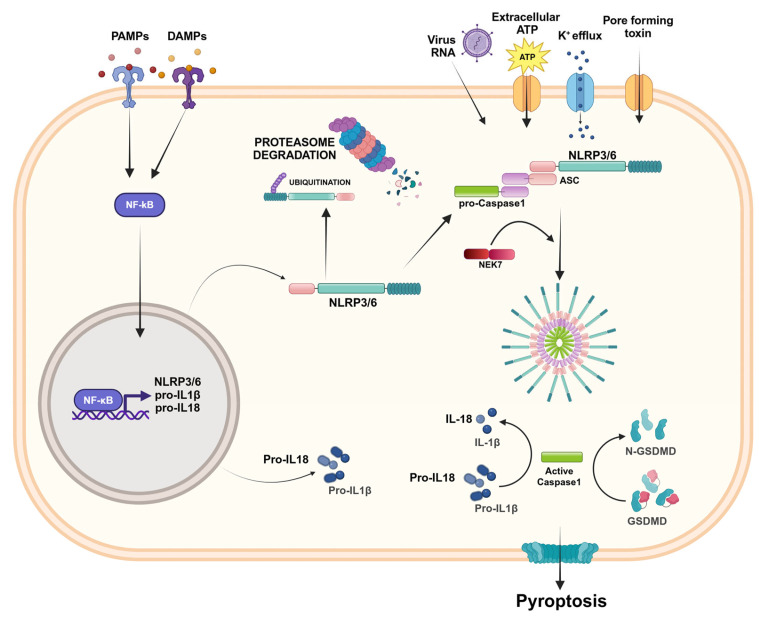
NLRP3/6 inflammasome activation. The activation of the NLRP3/6 inflammasome involves a two-step process. The first step, called “priming signal”, consists of the recognition of PAMPs or DAMPs, which results in NF-κB activation and the overexpression of inflammasome components. The second step, called “activation signal”, is provided by RNA viruses, extracellular ATP, the K^+^ efflux, or pore-forming toxins that lead the inflammasome assembly and oligomerization. NEK7 is a regulator that interacts with NLRP3 and mediates inflammasome assembly. The inflammasome complex activates caspase-1, which activates IL-1β and IL-18 and induces pyroptosis. The activation of the NLRP3 sensor can be regulated by post-transcriptional mechanisms and, in the absence of inflammatory stimuli, can be degraded by proteasomes. Created in BioRender.com.

To date, the NLRP3 inflammasome has been the most frequently studied inflammasome. NLRP3 consists of a cytosolic protein expressed by different types of cells, including immune cells, epithelial cells, microglia, and neurons, and promotes inflammatory signaling through the activation of caspase-1, the release of pro-inflammatory cytokines IL-1β and IL-18, and cell death by pyroptosis [21,22,23]. The activation of the NLRP3 inflammasome is related to PAMPs, DAMPS, and endogenous and exogenous stimuli, and involves a two-step process. The first step, or priming signal, corresponds to recognition of the TLR ligands, which results in the activation of a nuclear factor kappa-light-chain-enhancer of activated B cells (NF-κB) and the overexpression and post-translational modification of inflammasome components; the second step, or activating signal, triggered by extracellular ATP, K^+^ efflux, pore-forming toxins, and viral nucleic acids, promotes inflammasome assembly and oligomerization, caspase-1 activation, and the release of active IL-1β and IL-18 [21,22,23]. NLRP3 inflammasome activation is also controlled by regulators. In particular, never in mitosis A (NIMA)-related kinase 7 (NEK7) interacts with NLRP3 sensors and mediates the assembly and activation of the NLRP3 inflammasome downstream of the K^+^ efflux [24]. The activation of the NLRP3 inflammasome can also be regulated by post-transcriptional mechanisms (including phosphorylation, ubiquitination, SUMOylation, acetylation, and miRNA regulation) that affect NLRP3 as well its downstream effector molecules [25,26] (Figure 3).

The NLRP3 inflammasome plays an important role in regulating multiple inflammatory-related diseases, metabolic disorders, autoimmune diseases, and cancer [13,27,28]. In the gut, the NLRP3 inflammasome has a role in the host defense since it regulates intestinal homeostasis, controlling the integrity of the intestinal epithelium and modulating immune responses correlated to microbiota. However, several studies have reported controversial results on the role of NLRP3 in intestinal inflammation. Opposing functions have been identified, since some studies reported that IL-1β and IL-18 production contributes to intestinal inflammation; meanwhile, other reports have suggested that the production of IL-1β and IL-18 induced by the NLRP3 inflammasome protects against colitis and colitis-associated tumorigenesis [29,30,31].

Also in cancer, several points of evidence have shown the anti-tumorigenic and pro-tumorigenic effects of NLRP3 [28]. Deficiencies in different components of the NLRP3 inflammasome can lead to increased colitis and colon cancer; hence, the inflammasome can operate as a suppressor of inflammation and tumor progression [32]. Conversely, the NLRP3 inflammasome also has a pro-tumorigenic effect, since it is involved in proliferation, survival, metastasis, angiogenesis, and immunosuppression [28,33].

### 2.3. NLRC4

**Figure 4:**
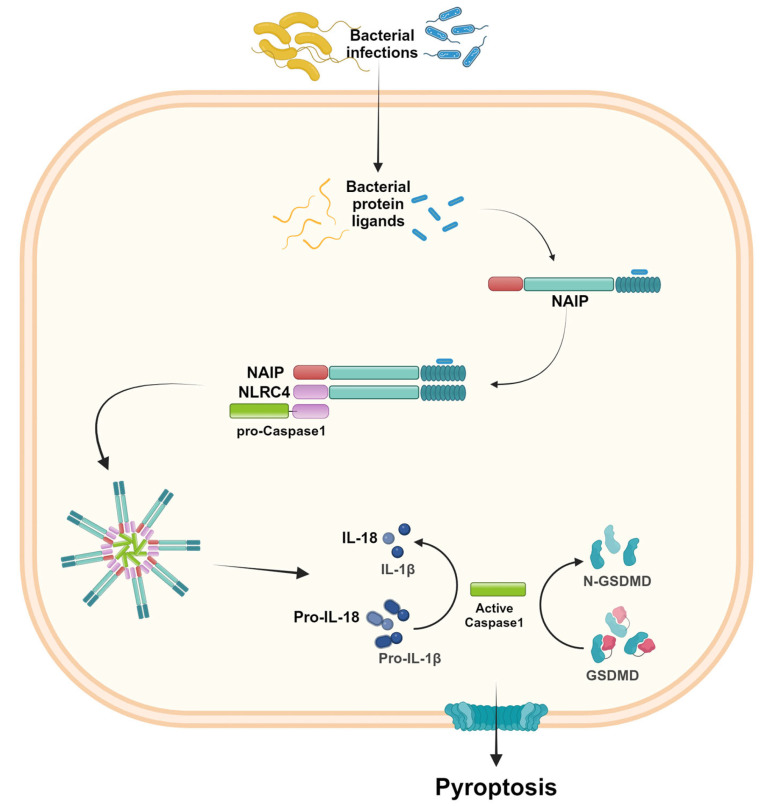
NLRC4 inflammasome activation. The presence of specific bacterial protein ligands is recognized by the NLR family of apoptosis-inhibitory proteins (NAIP). The NAIP-ligand complex recruits NLRC4, which therefore binds pro-caspase-1 and induces inflammasome oligomerization. As a result, the active caspase-1 induces IL-1β and IL-18 activation and release, as well as pyroptosis. Created in BioRender.com.

NLRC4 was originally identified as a pro-apoptotic protein due to its structure homology with apoptotic protease-activating factor-1 (APAF1) and its ability to activate caspase-1 [34]. Later, it was renamed and considered to be a member of the NLR family, owing to the presence of the CARD domain in the amino-terminal portion [35]. The NLRC4 inflammasome is assembled in response to bacterial infections, and the activation requires the recognition of specific bacterial protein ligands by the NLR family of apoptosis-inhibitory proteins (NAIP) [4,36]. The NAIP-ligand binding recruits NLRC4 and induces its oligomerization, influencing the functional NAIP-NLRC4 inflammasome assembly [36]. Like other inflammasomes, NLRC4 activation leads to IL-1β and IL-18 release and pyroptosis. However, through its CARD-CARD domain, it is able to activate caspase-1 without requiring the adaptor protein ASC [36] (Figure 4). The NAIP/NLRC4 inflammasomes may exhibit functions in distinct cell types. Most studies show the function of NAIP/NLRC4 inflammasomes in hematopoietic cells, mainly macrophages and dendritic cells. However, some evidence described NLRC4’s function in enterocytes [4,36,37].

Several studies have shown the critical role of NLRC4 in the host’s defense against bacterial infections [4,35]. The alteration of intestinal microbiota may contribute to ulcerative colitis progression. For instance, Desulfovibrio vulgaris (DSV), a common bacterial species present in the gut, is augmented in microbial dysbiosis and inflammatory conditions. An increase in DSV was observed in the feces of patients with UC, and was associated with disease activity. In addition, in a Dextran Sulfate Sodium (DSS)-induced colitis mouse model, DSV and its flagellin enhanced intestinal inflammation and induced significant macrophage pyroptosis, promoting NAIP/NLRC4 inflammasome activation [38]. Evidence for the involvement of NLRC4 in the pathogenesis of IBD is still contradictory. The lack of NLRC4 was reported to induce more severe inflammation and tissue damages, indicating its crucial role in protecting the gut [39]. In contrast, the absence or reduced activity of NLRC4 was found to make no difference to the severity of inflammation after DSS administration compared to the severity in wild type (WT) mice [40]. Moreover, NLRC4 plays an important role in human autoinflammatory disease, since gain-of-function mutations in the NLRC4 gene were identified as promoting spontaneous inflammasome activation [4,35].

The involvement of NLRC4 inflammasomes in tumorigenesis was also investigated. In patients affected by CRC, the gene expression of NLRC4 resulted in a decrease in the tumor portion compared to the adjacent normal tissue, and its lower expression was related to lymph node metastasis. In addition, the expression of NLRC4 was associated with overall survival in CRC patients, indicating that it can be a potential prognostic marker for predicting the survival rate of patients [41]. Furthermore, NLRC4 appears to play a protective role in the development of colon cancer. In fact, the deletion of genes encoding NLRC4 or NAIP increased the susceptibility of mice to the development of colitis and CRC associated with colitis [40,42]. In an azoxymethane (AOM)-DSS mice model with reduced NLRC4 activity and deficient for caspase-1, a significant increase in tumor development and aggressiveness was observed compared to the increase seen in wild type mice [40]. These findings suggest the protective activity of NLRC4/NAIPs against cancer development by promoting the suppression of colonic tumorigenesis.

### 2.4. NLRP6

NLRP6 is structurally similar to NLRP3 since it consists of a PYD N-terminal domain, an NBD central domain, and an LRR in the C-terminal portion (Figure 3). In the absence of inflammatory stimuli, the NLRP6 inflammasome maintains an auto-inhibitory conformation. In response to appropriate stimuli, such as viral and bacterial infections, it forms a complex with ASC and caspase-1 [43,44]. NLRP6 is a key regulator of intestinal homeostasis since it plays a critical role in regulating the host’s defense in intestinal epithelial cells and innate immune signaling in myeloid cells. It is able to recognize bacterial products and/or cell damage and integrity in the epithelium [45]. The NLRP6 inflammasome is highly expressed in the small and large intestine, and its expression has been demonstrated both in intestinal epithelial cells and goblet cells, as well as in immune cells [45].

The absence of NLRP6 results in the alteration of the gut microbiota, which leads to severe colitis [44,46]. In fact, NLRP6 deficiency was demonstrated to cause defective mucin secretion by goblet cells, which consequently triggered a greater susceptibility to persistent infection [47]. The aberrant gut microbial community also increased susceptibility to colitis-associated cancer in NLRP6 inflammasome-deficient mice after AOM/DSS administration, showing the critical activity of NLRP6 for protection against inflammation-related colon tumorigenesis [44,48].

### 2.5. AIM2-like Receptors

ALRs are cytosolic PRRs that detect exogenous nucleic acids during viral infection [3]. ALRs are structurally related proteins that generally act as intracellular DNA sensors. These sensors activate innate immune responses in the presence of DNA in the cytosol of infected or stressed cells. The ALR family includes the AIM2, IFNγ-inducible protein 16 (IFI16), pyrin and HIN domain family member 1 (PYHIN1, also called IFIX), and myeloid cell nuclear differentiation antigen (MNDA). ALRs are characterized by a PYD- and a DNA-binding domain hematopoietic IFN-inducible nuclear protein with 200 amino acids (HIN-200) [49] (Figure S1).

AIM2, identified for the first time in melanoma cell lines, was the first ALR member to be characterized in the innate immune system [50]. AIM2 is expressed in cytosol and consists of a sensor for double-strand DNA (dsDNA). This inflammasome is able to recognize viral and bacterial DNA, but also self-DNA distributed in the cytoplasm [51]. The binding of the HIN-200 domain with DNA relieves it from an autoinhibitory state and leads to an inflammasome complex assembly by ASC recruitment through its interaction with PYD, caspase-1 activation, and IL-1β and IL-18 release [51,52,53]. Moreover, as a protective action for the host, AIM2 inflammasome assembly can lead to PANapoptosis [54]. In CRC, AIM2 expression is reduced, and its role as tumor suppressor has been documented. A dysbiotic intestinal microbiota increased tumorigenesis in mice lacking AIM2, and stem cells tended to undergo uncontrolled proliferation [55].

Furthermore, AIM2 limited the proliferation of CRC cells, inhibiting cell viability, acting on the cell cycle by blocking cells at the G1/S transition level and promoting apoptosis via the phosphatidylinositol 3-kinase (PI3K)/protein kinase B (AKT)/P38 mitogen-activated protein kinase (MAPK) signaling pathways [41,56,57].

In humans, also IFI16, IFIX, and MNDA are included in the ALR family, while at least 13 members have been identified in mice. IFI16 contains two HIN domains for DNA binding, and it is a crucial contributor to the inflammatory response, since, after binding with viral DNA, it induces a strong expression of interferon-β (IFN-β) in human monocytes [49,50,51,52,53]. In addition, IFI16 is able to induce inflammasome complex activation in response to herpes virus infection and cell death of T cells via pyroptosis in HIV infection [58]. However, two members of the ALR family, IFIX and MNDA, have not yet been completely studied.

### 2.6. Pyrin Inflammasome

Pyrin, also knowns as Marenostrin (TRIM 20), is a large cytosolic protein mainly expressed in innate immune cells. Pyrin is composed of the N-Terminal PYD, which binds the adapter protein ASC; the central region consists of a zinc finger domain (B-box) and an α-helical coiled-coil domain (CC) necessary for correct protein oligomerization, as well as a C-terminal domain (B30.2/SPRY) [59]. The expression of pyrin inflammasomes can be influenced by LPS and cytokines (IFN-γ, TNF-α, IL-4, and IL-10), and, upon activation, pyrin recruits the adaptor protein ASC, leading to the activation and secretion of IL-1β and IL-18 and pyroptosis via GSDMD [59] (Figure S2).

To date, the physiological as well the pathological role of pyrin in the gastrointestinal tract is still unclear. However, there is evidence that the expression of the pyrin gene was considerably increased in IBD patients, and mutations of this gene are associated with a greater susceptibility to and severity of IBD [60]. Moreover, expression of the pyrin gene was found to be significantly higher in colon cancer [61].

## 3. Role of Inflammasomes in Intestinal Diseases

Inflammasomes are an important component of the innate immune system which recognizes pathological infections and endogenous danger signals and triggers an effective and rapid immune response. However, dysregulated inflammasomes activation is implicated in the pathogenesis of a variety of intestinal diseases. Over the years, the contribution of inflammasomes in the pathogenesis of several inflammatory diseases, such as IBS, IBD, Celiac Disease, and CRC, has been demonstrated.

### 3.1. Irritable Bowel Disease

IBS is a chronic functional bowel disorder related to an altered bowel function [62]. The pathophysiology is complex and far from well understood. In any case, IBS is considered to be a multifactorial disorder in which several mechanisms are involved, including dietary, altered visceral sensitivity, altered gut–brain axis, bowel motility and secretory dysfunctions, and somatic and psychiatric comorbidities [62]. Furthermore, microbial dysbiosis, impaired mucosal barrier functions, immune activation, and the release of immune mediators have been associated with IBS [63]. The development of IBS can be caused by acute gastrointestinal infections (bacterial, viral, and protozoal), thus causing post-infection IBS (PI-IBS) [64].

Studies have reported the involvement of inflammasomes in IBS. In a PI-IBS mice model, it was demonstrated that the expression of NLRP3, ASC, and caspase-1 was significantly increased compared to that of the control groups both in the chronic and acute inflammation phases [65]. In fact, IBS patients with an intestinal flora disturbance present an altered NLRP3 expression that further aggravates the intestinal inflammation, indicating the role of NLRP3 in the regulation of intestinal flora and maintenance of intestinal homeostasis [66]. The alteration of intestinal microbiota triggered by stress-induced intestinal inflammation influencing NLRP6 inflammasome signaling in the intestinal mucosa of IBS patients. In fact, some researchers have reported that the reduction in NLRP6 expression and the alteration of microbial intestinal composition caused intestinal inflammation [67,68].

### 3.2. Celiac Disease

Celiac disease is one of the most common immune-mediated chronic enteropathies, characterized by an immune response to gluten in genetically predisposed subjects [69,70]. Celiac disease is an autoimmune disease that only occurs after exposure to the exogenous trigger. It is characterized by a genetic predisposition linked to human leukocyte antigen (HLA)-DQ2 and -DQ8 and tissue transglutaminase autoantigen (tTG), an improper innate and adaptive inflammatory response that can target enterocytes leading to loss of function of the intestinal barrier and an altered intestinal microbiome [69,70].

In patients with Celiac disease, the involvement of products downstream of the inflammasome activation, such as IL-1β and IL-18, was demonstrated [71]. In peripheral blood mononuclear cells (PBMC) and monocytes from celiac patients, the pepsin digest of wheat gliadin fraction (PDWGF) induces the secretion of IL-1α, the activation of caspase-1, and the secretion of mature IL-1β and IL-18 dependent on the NLRP3 inflammasome [72]. Moreover, in the intestine, the p31–43 peptide from a-gliadin may induce an innate immune response that consequently triggers pathological intestinal inflammation through NLRP3 inflammasome activation [73]. In addition, the pyroptosis activated by inflammasome signaling was correlated to colon tissue damage present in Celiac disease patients [74]. In addition, the polymorphisms in NLRP3 and NLRP1 genes were associated with Celiac disease, suggesting their involvement in the predisposition to the disease. Single nucleotides polymorphisms (SNPs) in the NLRP1 gene may lead to an increased inflammasome activation, contributing, together with NLRP3, to a predisposition to Celiac disease. In fact, an abnormal activation of NLRP1 and/or NLRP3 inflammasomes results in an altered activation of IL-1β and NF-κB, contributing to the lack of immune tolerance common in Celiac disease [75].

### 3.3. Inflammatory Bowel Disease

IBD is a group of heterogeneous diseases characterized by chronic inflammation of the gastrointestinal (GI) tract that occurs in the form of recurrent episodes alternating with remission periods. Based on the symptoms, disease location, and histopathological characteristics, IBD is classified as CD or UC. Although the etiology is still uncertain, IBD is a multifactorial disorder in which genetic susceptibility, the impaired intestinal barrier, and the aberrant immune response directed at the intestinal microbiota are all involved [76].

NLRs have been shown to contribute, directly or indirectly, to IBD pathogenesis [4]. In IBD patients, the levels of NLRP3 inflammasome components increase and are related to the disease’s severity [77]. In addition, genome-wide association studies revealed that the deregulation of NLRs was associated with IBD development in genetically predisposed individuals. In fact, polymorphisms in the NLRP3 gene and inflammasome effectors were associated with an increased susceptibility to IBD [78,79,80]. Studies on experimental models of colitis report contradictory results regarding the role of NLRP3 in IBD pathogenesis. Some works show that NLRP3, as well as caspase-1, play crucial roles in regulating immune response and maintaining intestinal homeostasis, and their deletion causes a greater susceptibility to DSS-induced colitis and increased intestinal epithelial damage, supporting the protective qualities of NLRP3 [29,31]. On the contrary, the study conducted by Siegmund and co-workers reported that a caspase-1 deficit in DSS-induced colitis mice had protective effects on the evolution of the disease, related to the limited production of IL-1β and IL-18 [81]. The overproduction of IL-18 was responsible for a significant immune infiltration in intestinal mucosa [82]. Bauer and collaborators demonstrated a critical role of NLRP3 inflammasomes in intestinal inflammation in the DSS colitis model, since NLRP3-deficient mice developed mild colitis, unlike WT mice, and the levels of pro-inflammatory cytokines in colonic tissue were reduced [30].

NLRP3 is the inflammasome most closely studied in relation to IBD pathogenesis. However, other inflammasomes have also been investigated to assess their contribution to IBD. Some evidence describes the role of NLRC4 in protecting the gut, since the absence of this sensor caused colonic epithelial injury in DSS-induced colitis mice [39], whereas another work reported that the severity of colitis was not affected by NLRC4 deficiencies [40]. Furthermore, mutations in NLRC4 were associated with an increased susceptibility to UC development [83]. Despite this, the involvement of NLRC4 in the pathogenesis of IBD is still unclear.

NLRP1 plays a role in regulating microbiome composition and has protective effects on colitis, while its absence gives rise to the proliferation of pathogenic bacteria that induce dysbiosis and consequently exacerbate intestinal inflammation and impair the function of the intestinal barrier [20,84]. Furthermore, NLRP1 polymorphisms were associated with colonic inflammation in CD patients [85].

NLRP6 expression is elevated in intestinal tissue and plays a crucial role in intestinal homeostasis. NLRP6 reduces inflammation and carcinogenesis, and regulates the restoration of tissue damage. The absence of NLRP6 in experimental colitis results in a greater susceptibility to the development of the disease, determining the impairment of the intestinal microbiota, severe intestinal inflammation, and the inability to restore epithelial barrier integrity [43,45,46,86]. Moreover, NLRP6 deficiencies predisposed the host to colitis-associated tumor growth [44,86]. However, other findings did not show an impact of NLRP6 on the gut microbiota composition; therefore, the role of NLRP6 in intestinal inflammation is not yet fully clarified [87,88].

The AIM2 inflammasome has also a protective role in the intestine, since it modulates gut homeostasis. The absence of AIM2 affected caspase-1 activation and the production of IL-1β and IL-18, whilst also making mice highly susceptible to DSS-induced colitis associated with microbial dysbiosis [89]. A subsequent study found that the release of IL-18 triggered the upregulation of the IL-22-binding protein (IL-22BP) and antimicrobial peptides. Hence, the suppression of IL-22BP related to AIM2 deletion caused the loss of the STAT3-dependent antimicrobial peptides (AMPs) Reg3β and Reg3γ, which promoted dysbiosis-linked colitis [90].

### 3.4. Colorectal Cancer

CRC is the second cause of cancer-related death and the third most commonly diagnosed form of tumor in the world. CRC development is a multiphase process that develops from a progressive accumulation of genetic and epigenetic alterations, genomic, and/or epigenetic instability [91].

Inflammation plays an important role in tumor growth, since an imbalance in the influx of inflammation-associated cytokines and chemokines determines a favorable tumor microenvironment (TME) that promotes tumor progression [92]. Over the years, studies have shown that altered inflammasome signaling contributes to inflammation and colorectal tumorigenesis [4]. NLRP1, NLRP3, NLRC4, NLRP6, and AIM2 have been demonstrated to influence the pathogenesis of cancer by modulating innate and adaptive immune responses, cell death, and proliferation [41].

The expression of NLRP3 is higher in human CRC tissues compared with non-cancerous tissues; it is correlated with TNM staging and is also required for epithelial–mesenchymal transition (EMT) [33,93]. In fact, the inhibition of NLRP3 expression reduced the migratory and invasive capacities of cancer cells, whilst also inverting the mesenchymal condition by reducing vimentin and Matrix metalloproteinase-9 (MMP9) expression and increasing E-cadherin expression [33,93]. In CRC mice models, the protective role of the NLRP3 inflammasome was shown, and its deficiency made the mice more susceptible to colitis and colitis-associated colorectal cancer induced by AOM/DSS [32].

NLRP6 plays a protective role in intestinal inflammation and tumorigenesis. There is evidence that mice lacking NLRP6 showed greater CRC development, resulting in the inability to reduce inflammation and repair epithelial damage as well the proliferation and migration processes [44,86]. Moreover, the promotion of tumorigenesis linked to the lack of NLRP6 was associated with increased inflammation due to aberrant microbiota induced through the production of CCL5 and the increased proliferation of epithelial cells triggered by the production of IL6 [48].

The NLRP1 inflammasome also plays a protective role in the development of colitis-associated CRC, which is associated with the release of the effector cytokines IL-1β and IL-18. A study conducted using an AOM-DSS mouse model reported that mice deficient in NLRP1 had increased morbidity, inflammation, and tumorigenesis, as well as low levels of IL-1β and IL-18 compared to WT mice. By restoring the levels of effector cytokines, the severity of colitis in Nlrp1b^−/−^ mice was reduced [20].

The ASC adaptor protein, as well as the caspase-1 effector, may be involved in tumor progression. Mice lacking ASC and caspase-1 showed an increased tumor burden related to attenuated levels of IL-1β and IL-18 in the site of the tumor [32]. Moreover, the methylation status of the ASC gene could be used as a marker for cancer prognosis, since ASC is epigenetically silenced by aberrant methylation of its CpG islands in many tumor cells, suggesting its potential role as a tumor-suppressor gene. In a work conducted by Hong and colleagues, they discovered that ASC-deficient colon cancer cells underwent cell death in response to genotoxic stress, supporting the concept that the methylation status of the ASC gene could be used for cancer prognosis [94]. In the absence of caspase-1 or NLRC4 activity, the epithelial cells exhibit an enhanced tumorigenesis. In Caspase-1–deficient mice, enhanced tumor formation resulted in intrinsic changes in epithelial gene-expression programming affecting proliferation and apoptosis. This increased tumorigenesis was also mediated by NLRC4 inflammasomes [40].

Cytokine production as a result of inflammasome activation was shown to be protective against colorectal tumorigenesis. The lack of both the IL-1β and IL-18 receptors causes a greater susceptibility to DSS-induced colitis and CRC development [95]. Zaki and co-workers demonstrated that the lack of NLRP3 inflammasome components results in the development of inflammation and significant progression of CRC in an AOM/DSS mouse model. Mice with NLRP3 and caspase-1 deficiency subjected to AOM/DSS showed a dramatically increased tumor burden in the colon due to reduced IL-18 levels that altered the production and activation of the tumor suppressors IFN-γ and signal transducer and activator of transcription 1 (STAT1) [32]. Moreover, in Nlrp1b^−/−^ mice, an increase in inflammation and tumorigenesis was observed, followed by attenuated levels of IL-1β and IL-18 [20]. Similarly, pyrin-deficient mice have shown a greater susceptibility to colon tumor formation compared to WT mice in response to AOM/DSS, also showing a consequent defect in IL-18 production [96].

Some studies have been conducted to demonstrate the role of AIM2 inflammasome components in CRC carcinogenesis. Over the years, studies on AIM2 showed its role as a tumor suppressor, showing that a reduced expression of the AIM2 gene was correlated with cancer progression and poor prognosis in CRC [41,97]. In vitro studies on HCT116 cell lines show that the restoration of AIM2 levels block cell proliferation in the late S-phase. Despite this, these cells show an aggressive phenotype, expressing genes associated with invasion [98]. AIM2 was also involved in the suppression of the EMT and cell invasion, in the inhibition of cell viability, and in the apoptosis of CRC cells through the suppression of the PI3K/AKT pathway [99]. In a study on AIM2-deficient mice, higher tumor development in the colon was observed following AOM/DSS treatment that induced AKT activation [100].

## 4. Strategies to Inhibit the Inflammasomes Pathways

Considering the clear contribution of inflammasomes to the onset and progression of various inflammation-related diseases, therapeutic strategies that target the inflammasome signaling pathways offer considerable therapeutic promise in the treatment of inflammatory diseases.

**Figure 5:**
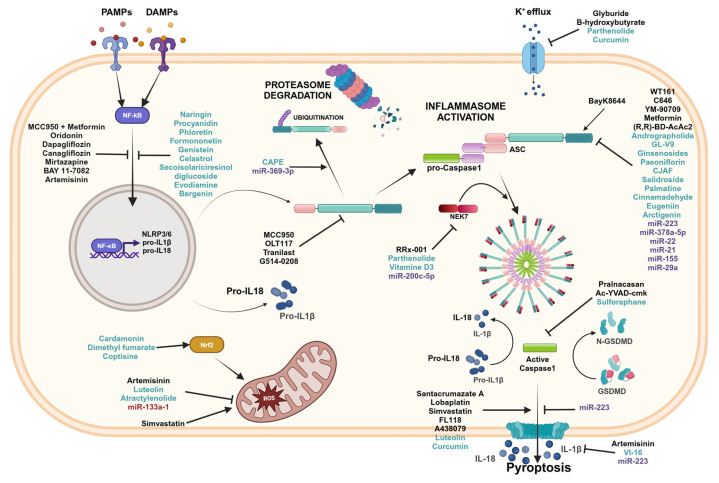
Inflammasomes inhibitors. Graphical representation of the inflammasome pathways and inhibitors with related targets. Black font corresponds to pharmacological compounds, green font corresponds to natural compounds, and purple font corresponds to miRNAs. Created in BioRender.com.

To date, several molecules that directly target the sensors, as well as other components of the inflammasome complexes and downstream effector molecules have been identified (Figure 5). Here, we list the known therapeutic inhibitors of the inflammasomes pathways and their respective intestinal disease targets.

### 4.1. Pharmaceutical Inhibitors

Several pharmacological inhibitors that target the inflammasome sensors have been reported (Table 1). Many compounds that directly interact with NLRP3 inflammasomes binding the NACHT domain include MCC950, OLT117, Tranilast, and G514-0208.

MCC950 is the best characterized and most potent and selective small molecule inhibitor that targets the NLRP3 inflammasome. MCC950 is able to block canonical and non-canonical NLRP3 activation by directly binding the NLRP3 NACHT domain [101]. In a spontaneous chronic colitis mouse model, MCC950 reduced the severity of chronic colitis through the inhibition of NLRP3 inflammasome activation. Consequently, the selective inhibition of NLRP3 by MCC950 decreased IL-1β secretion [102]. Loss of function of the intracellular bacterial NOD2 sensor is a risk factor for the development of CD. Even in the absence of NOD2 signaling, the inhibition of the NLRP3 pathway can prevent intestinal inflammation. In fact, it has been reported that MCC950 reduced DSS-induced intestinal inflammation in a NOD2-sensor-deficient mouse model [103]. Immunity-related GTPase M (IRGM) is a protein that acts as a negative regulator of the NLRP3 inflammasome activation since it physically interacts with the components of inflammasomes, preventing assembly and mediating the autophagic degradation of inflammasome components. It has been demonstrated that treatment with MCC950 improved colitis in mice with an IRGM deficiency, blocking NLRP3 inflammasome activation [104].

OLT117, a β-sulfonyl nitrile compound, is a selective inhibitor for NLRP3 that is able to inhibit both canonical and non-canonical NLRP3 inflammasome activation by directly binding NLRP3 on the NACHT domain. The administration of OLT1177 showed a greater efficiency during the acute inflammatory phase of DSS-induced colitis than during the recovery phase [105].

Tranilast (TR) is an analog of the tryptophan metabolite, which has a protective effect in IBD. TR showed an inhibitory effect on the NLRP3 inflammasome in macrophages by binding directly to the NACHT domain and thus inhibiting the assembly of NLRP3 [106]. TR reduced experimental colitis, decreasing the expression of the pro-inflammatory cytokines and increasing anti-inflammatory cytokine levels [107,108]. Moreover, TR inhibited tumor growth and angiogenesis and increased tumor necrosis by targeting transforming growth factor beta (TGF-β) in colon cancer [133]. Moreover, intragastric administration of TR in rat models with IBS inhibited visceral hypersensitivity and colon permeability in a dose-dependent manner by suppressing the NLRP3 inflammasome [109].

A small-molecule drug, G514-0208, is an inhibitor with a tetrahydroquinoline core which selectively inhibited the NLRP3 sensor by NACHT domain binding. This molecule inhibited NLRP3 assembly and activation, reducing experimental colitis [110].

Other inhibitors control inflammasomes activation by regulating the NF-κB activity. The protective effect of MCC950, in combination with metformin, interrupted the priming signal needed for NLRP3 inflammasome activation via TLR4/NF-κB signaling and led to autophagy induction by AMP-activated protein kinase (AMPK) [111]. Other antidiabetic drugs, such as Dapagliflozin and Canagliflozin, have been reported to have a coloprotective effect against UC via NLRP3 signaling inhibition, preventing both NLRP3 inflammasome priming and the activation step, IL-1β and IL-18 release, and pyroptosis [112,113]. Oridonin (Ori) is a bioactive constituent of a medicinal herb with anti-inflammatory properties and may be considered a potential candidate for the treatment of IBD. In fact, in a colitis mouse model, it was found that Ori inhibited the activation of NF-κB and suppressed the overexpression of pro-inflammatory cytokines [114]. Mirtazapine (MRT) is a well-established antidepressant drug that demonstrated a favorable effect in UC treatment, decreasing the expression of NLRP3 inflammasome components [115]. BAY 11-7082 is a potent inhibitor of the NF-κB pathway that also selectively suppresses the activation of NLRP3 in macrophages [116]. BAY 11-7082 demonstrated a therapeutic effect also in IBS, significantly reducing the pathological signs of disease by inhibiting NLRP3 inflammasome activity and NF-κB translocation, thus reducing the release of inflammatory mediators [66].

Artemisinin and its derivatives exhibit anti-inflammatory properties in anti-inflammatory diseases. Artemisitene significantly reduced the inflammatory response in DSS-induced ulcerative colitis, inhibited the production of reactive oxygen species (ROS), and blocked the activation of NLRP3 inflammasome, inhibiting caspase-1 cleavage, and, hence, IL-1β production. Moreover, it can also inhibit IL-1β secretion mediated by NLRC4 and the AIM2 inflammasome [117]. Dihydroartemisinin (DHA) is another compound, derivative of artemisinin, which protects against acute colitis via suppressing NLRP3 inflammasome activation and NF-κB and p38 MAPK signaling [134].

One of the stimuli that led to inflammasome activation is K^+^ efflux [21,23]. Glyburide and β-hydroxybutyrate are two compounds that regulate inflammasome activation in this step. Glyburide, an antidiabetic drug used to treat type 2 diabetes, can prevent the activation of the NLRP3 inflammasome and decrease IL-1β release in immune cells through the inhibition of K^+^ efflux [118]. In IL-10-deficient mice, Glyburide suppressed NLRP3 inflammasome activation, acting both preventively and by improving ongoing colitis [77]. β-hydroxybutyrate (BHB) is a ketone metabolite that can act as an important immune cell regulator since it induces NLRP3 inflammasome inhibition by suppressing K^+^ efflux and reducing ASC oligomerization [119]. Its integration with exogenous BHB may provide a useful therapeutic approach for IBD treatment. Recently, it was discovered that treatment with exogenous BHB mitigated experimental colitis in rat model colitis through the inhibition of NLRP3 inflammasome activation, resulting in a low release of IL-1β and IL-18 and attenuated intestinal epithelial damage, as well as in the restoration of the intestinal epithelial barrier. Furthermore, BHB downstream mitigated apoptosis and pyroptosis processes by inactivating caspase-3 and NLRP3/GSDMD, respectively [120]. Another chemical strictly related to BHB is (R,R)-BD-AcAc2, a ketone ester able to increase the serum levels of BHB, which may offer another potential therapeutic approach in IBD. Notably, (R,R)-BD-AcAc2 improved experimental colitis, targeting the priming and activation of the NLRP3 inflammasome and its downstream signaling molecules, inhibiting pyroptosis and inducing autophagy [121].

An essential mediator of NLRP3 activation is NEK7, which acts downstream of K^+^ efflux to regulate NLRP3 oligomerization and activation [135]. RRx-001 is a highly selective and potent NLRP3 inhibitor since it blocks the assembly and activation of the NLRP3 inflammasome by ensuring NLRP3-NEK7 interaction. Consequently, RRx-001 ameliorated the inflammatory conditions and attenuated symptoms in experimental colitis [122].

Histone deacetylase 6 (HDAC6) was shown to be important in NLRP3 inflammasome assembly and activation [136]. WT161, a specific inhibitor for HDAC6, alleviated colonic inflammation and intestinal injury in DSS-induced colitis mice via NLRP3 inflammasome suppression. WT161 decreased the expression of NLRP3, suppressed ASC oligomerization, and reduced the levels of pro-inflammatory factors [123]. C646 is an inhibitor of histone acetyltransferase p300, which ameliorates inflammation in a DSS-induced colitis model by affecting NLRP3 inflammasome assembly and activation. Xu and co-workers reported that C646 impaired NLRP3 inflammasome activation through the suppression of ASC oligomerization, inhibiting interactions with NLRP3 and ameliorating experimental colitis [124].

The activation of caspase-1 by the NLRP3 inflammasome induces pathological mechanisms in IBD, and its inhibition could offer a useful strategy for IBD treatment. In fact, Bauer and co-workers reported that the administration of Pralnacasan, a potent and non-peptide inhibitor for caspase-1, prevented DSS-induced colitis [30]. Another Caspase-1-specific inhibitor, namely Ac-YVAD-cmk, suppressed intestinal inflammation and reduced the severity of colitis in Il10-deficient mice [127].

Pyroptosis has a significant anti-tumor effect in CRC. Inducing pyroptosis is considered to be a promising therapeutic strategy for CRC patients since it could overcome resistance to conventional chemotherapy drugs and enhance immunotherapy, and, therefore, treatment outcomes [137]. Loss of NLRP3 expression in CRC limits pyroptosis, affecting the efficacy of antitumor therapy. Guan and co-workers showed that the overexpression of HDAC2 in CRC silenced the NLRP3 inflammasome, limiting GSDMD-mediated pyroptosis. The inhibition of Histone deacetylase 2 (HDAC2) by the selective histone deacetylase inhibitor Santacruzamate A, in combination with 5-Fluorouracil (5-FU) and regorafenib antitumor agents, activated NLRP3/GSDMD-mediated pyroptosis [128]. Yu and colleagues reported that, in CRC cell lines treated with lobaplatin, an anti-neoplastic agent, reduced the cell viability and induced pyroptosis by triggering GSDME cleavage in a dose-dependent manner [129]. Simvastatin is indicated for the treatment of hyperlipidemia, although there is evidence that, in cancer, Simvastatin affected cell proliferation and angiogenesis and acted against drug resistance [138]. It was reported that simvastatin inhibited the cell proliferation and viability of colon cancer cells by inducing pyroptosis. The administration of Simvastatin enhanced the assembly and activation of the NLRP3 inflammasome, induced the release of IL-1β and IL-18 effectors, and mediated GASDMD activation, and hence pyroptosis, through increased cellular ROS [130]. FL118 is an antitumor drug that showed greater antineoplastic effects in CRC [139]. Tang and colleagues reported that FL118 inhibited cell proliferation, the migration and invasion of colon cancer cells, and promoted pyroptosis by activating the NLRP3 inflammasome [131]. Similarly, A438079, a selective and competitive P2X7 receptor antagonist, affected CRC cell line proliferation, invasion, and migration, in addition to inducing apoptosis via the Bcl-2/caspase-9/caspase-3 pathway and pyroptosis through the NLRP3/caspase-1 pathway [132].

The TME plays a key role in cancer progression and invasion, since it allows for communication between tumor cells and other cell types present in the tumor stroma. The persistent intercommunication exposes the TME to cytokines, chemokines, and growth factors that support tumor growth [92]. The inhibition of these inflammatory processes could be used as a therapeutic target in CRC. In a study conducted using colon cancer cell lines, it was discovered that metformin can suppress inflammatory factors’ expression, regulate the inflammatory microenvironment, and limit the progression of CRC by inhibiting the expression of the NLRP3 inflammasome [125].

Transmembrane protein 176B (TMEM176B) is an immunoregulatory cation channel highly expressed in the tumor stroma in CRC patients, and it is associated with poor prognosis. TMEM176B in immune cells acts as a negative regulator of inflammasome activation, since overexpression of TMEM176B is associated with reduced NLRP3 and IL-1β expression. Segovia and co-workers demonstrated that the inhibition of TMEM176B expression with BayK8644 triggered inflammasome activation and consequently limited tumor growth. Furthermore, they reported that BayK8644 administration, in combination with immune checkpoint blockers, augmented the antitumoral effects [126].

### 4.2. Natural Compounds

Many natural compounds have anti-inflammatory properties and are able to influence the aberrant inflammation due to their ability to interfere with several steps leading to inflammasomes activation, assembly, and the resulting signaling pathways (Table 2).

Several molecules suppress the activation of NF-κB, preventing NLRP3 inflammasome activation. Naringin and Procyanidin, two powerful antioxidation flavonoids, alleviated disease activity in DSS-induced colitis models, suppressing the activation of NF-κB signaling and NLRP3 inflammasome activation [140,141]. Likewise, Phloretin, by inhibiting the NF-κB signaling pathway, suppressed NLRP3 inflammasome activation, reduced the release of pro-inflammatory cytokines, and maintained intestinal epithelial integrity [142].

Formononetin and Genistein are two isoflavone compounds that have protective effects on DSS-induced acute colitis, attenuating colon epithelial damage and reducing protein levels of the NLRP3 pathway by inhibiting NF-κB [143,144].

Also, Celastrol, Evodiamine (EVO), Bergenin, and Secoisolariciresinol diglucoside have the capacity to inhibit inflammasome activation via NF-κB. The significant therapeutic properties of Celastrol, EVO, and Bergenin have been demonstrated in IBD [145,146,147,174]. These natural compounds improved inflammatory conditions and colon tissue damage in a DSS-induced colitis mouse model by regulating the NF-κB signal and NLRP3 inflammasome, as well as the damage to the colon epithelial barrier, restoring the tight junction (TJ) architecture. Secoisolariciresinol diglucoside was shown to attenuate inflammatory conditions and reduce the release of pro-inflammatory cytokines by modulating the activation of both the NLRP1 inflammasome and the nuclear NF-κB pathway [148].

Another pathway that modulates activation of the NLRP3 inflammasome is NFE2-Like BZIP Transcription Factor 2 (Nrf2 signaling); in fact, the mediator Nrf2 is considered to be a positive regulator of NLRP3 activation [149]. The Nrf2-activating compounds, Cardamonin, Dimethyl fumarate (DMF), and Coptisine, are able to inhibit inflammasome activation. Cardamonin and DMF, having anti-inflammatory and antioxidant effects, improved colitis in a DSS-induced colitis mouse model through activation of the Nrf2 pathway, thus reducing the activation of caspase-1 and the release of effector molecules [149,150]. In the PI-IBS, Coptisine significantly increased the expression of Nrf2 that, in turn, modulates the protein levels of NLRP3, ASC, and caspase-1. This modulation ameliorated intestinal symptoms, decreasing the markers of oxidative stress and pro-inflammatory cytokines production in PI-IBS rats [151].

Luteolin, a flavonoid, exerted anti-inflammatory properties, decreasing ROS production, reducing NLRP3 inflammasome-related components’ expression, and promoting macrophage polarization towards M2 phenotypes [152,173]. In CRC, Luteolin increased the expression of caspase-1, GSDMD, and IL-1β, inhibiting the growth and proliferation of colon cancer cells and promoting pyroptosis [169]. Atractylenolide (ATR I) suppressed NLRP3 inflammasome activation in colitis-associated colorectal cancer via the inhibition of mitochondrial damage. Treatment with ATR I reduced cell viability and induced apoptosis in CRC cell lines and inhibited colonic neoplasms in the AOM/DSS mice model [153].

K^+^ efflux is the common trigger of inflammasomes activation [21,23]. Studies have shown that Parthenolide (PTL) not only inhibits NLRP3 inflammasome activation by suppressing K^+^ efflux, but also suppresses assembly of the NLRP3 inflammasome complex, affecting the NLRP3-NEK7 interaction, the oligomerization of NLRP3, and the NLRP3-ASC interaction. This, in turn, inhibits caspase-1 activation and pyroptosis [116,154]. Another polyphenol, Curcumin, is able to inhibit activation of the NLRP3 inflammasome to reduce the release of IL-1β and modulate the K^+^ efflux, resulting in intracellular formation of ROS and the release of Cathepsin B [155]. In CRC cell lines, Curcumin decreased viability and promoted NLRP3-mediated pyroptosis [170]. Vitamin D3 (VitD3) reduces inflammation in DSS-induced UC by suppressing NLRP3 inflammasome activation and preventing ASC oligomerization, thus inhibiting caspase-1 activation and IL-1β production. Moreover, VitD3 impairs NLRP3-NEK7 interactions, modulating pyroptosis [156].

A variety of natural products are able to suppress inflammasome component expressions, inhibiting their assembly and activation. Ginsenosides are bioactive metabolites derived from Panax ginseng which play an anti-inflammatory role in DSS-induced UC by suppressing NLRP3 inflammasome activation [157,158]. Salidroside (Sal), Palmatin, and Cinnamaldehyde (CA) act to alleviate the disease activity index (DAI) in DSS-induced colitis mouse models by downregulating NLRP3 inflammasome activation and restoring the expression levels of pro-inflammatory mediators [159,160,162].

In IBS, Paeoniflorin and Changji’an Formula (CJAF) inhibited the altered activation of the NLRP3 inflammasome pathway and re-established the impaired intestinal barrier functions, increasing the tight junction proteins [163,164].

Eugeniin, a polyphenol extracted from cloves and other plants, has anti-inflammatory properties preventing NAIP/NLRC4 inflammasome activation and thus UC development [38]. Qiao and colleagues reported that Arctigenin (ATG), a bioactive metabolite that possesses anti-inflammatory and anticancer activities, has a potential preventive application in reducing the risk of CRC in patients with UC. They showed that the inhibition of the NLRP3 inflammasome assembly and the consequent downregulation of IL-1β release by ATG reduced carcinogenesis in AOM/DSS-induced CAC mice [175].

Andrographolide (Andro) is a natural diterpenoid with antibacterial, anti-asthmatic, antiviral, and neuroprotective activities that attenuates the progression of colitis and tumor burden. Andro mitigates inflammation through inhibition of the formation and activation of inflammasome NLRP3 in macrophages, thus inhibiting the activation of caspase-1 and the secretion of IL-1β. Mitophagy triggered by Andro was responsible for the inhibition of inflammasome NLRP3 [166]. Furthermore, in CRC, Andro can be used in combination with chemotherapeutic agents. There is wide use of 5-fluorouracil (5-FU)-based chemotherapy in the treatment of CRC, and it was shown to significantly activate the NLRP3 inflammasome in myeloid-derived suppressor cells (MDSCs). Xu and colleagues reported that Andro, in combination with 5-FU, enhances the anticancer effects of treatment through the inhibition of 5-FU induced NLRP3 activation [167].

GL-V9 (5-hydroxy-8-methoxy-2-phenyl-7-(4-(pyrrolidin-1-yl)butoxy)4 H-chromen-4-one) is a synthesized flavonoid with strong anti-inflammatory and anti-tumor activities that is able to modulate the signaling pathway associated with the NLRP3 inflammasome. It has been demonstrated that GL-V9 triggers autophagy in macrophages via AMPK signaling, degrades the NLRP3 inflammasome, and attenuates DSS-induced colitis. Moreover, GL-V9 also reduces inflammation in colitis-associated colon cancer [168].

The activation of caspase-1, as well as the maturation and secretion of IL-1β downstream of NLRP1, NLRP3, NLRC4, and AIM2, could be inhibited by Sulforaphane (SFN) [171]. Due to its anti-inflammatory properties, SFN was analyzed in the treatment of IBD [176]. The inhibition of NLRP3 activation restored levels of IL-18 and IL-1β and ROS, thus reducing inflammation [172]. VI-16, a synthetic flavonoid compound, shows powerful anti-inflammatory effects, demonstrating its potential use for the treatment of IBD. In mice with DSS-induced colitis, VI-16 intervened by inhibiting NLRP3 inflammasome-related IL-1β release and reducing oxidative stress [173].

Caffeic acid phenethyl ester (CAPE) was shown to prevent CAC by inhibiting NLRP3 inflammasome post-transcriptionally. CAPE facilitated the binding of NLRP3 to ubiquitin molecules, promoting the ubiquitination and degradation of NLRP3 and contributing to the anticancer effect in the AOM/DSS mouse model [165].

### 4.3. microRNA

MicroRNAs (miRNAs) are highly conserved small non-coding RNA molecules (20–24 nucleotides long) that play a pivotal role in the regulation of gene expression post-transcriptionally. Their binding on complementary sequences of messenger RNA (mRNA) targets regulates gene expression, inhibiting or altering protein translation [177].

miRNAs may act as regulators of inflammasome signaling (Table 3).

One of the most closely studied miRNAs involved in inflammasomes activation in multiple steps is miR-223. miR-223 suppresses NLRP3 expression, reducing its activity and preventing its aberrant activation. However, miR-223 expression was high in the steady state, although it was not functional; thus, to inhibit NLRP3, it needs to reach a specific expression threshold [178]. In IBD, miR-223 inhibited NLRP3 expression and reduced IL-1β production, ameliorating colitis in a mouse model [182]. Moreover, an analysis of circulating miRNAs in the serum of UC patients highlighted that miR-223 was overexpressed, defining it as a possible biomarker and therapeutic target in the treatment of IBD [185]. Additionally, in macrophages, it was demonstrated that miR-223 inhibited the activation of NLRP3, and hence pyroptosis, via the miR-223/NLRP3 axis [183].

Two miRNAs upregulated in IBD and in IBS, miR-21 and miR-155, can be considered as potential therapeutic targets to modulate the mucosal immune response [186]. It was proven that the Cinnamaldehyde (CA) ameliorated DSS-induced colitis by reducing the expression levels of miR-21 and miR-155 in the colon, and, as a consequence, blocked the activation of the NLRP3 inflammasome [161]. miRNA-29a is another miRNA found to be increased in the colonic tissue of patients with IBS. It increased intestinal permeability by targeting the NF-kΒ-repressing factor (NKRF), a regulator of NLRP3 priming and activation. In IBS mice models, it was shown that paeoniflorin exercised protective effects, modulating the expression of miR-29a. As a result, NLRP3 inflammasome activation and tissue damage were significantly reduced [163].

ROS generation plays a critical role in the activation of NLRP3. Bandyopadhyay and co-workers investigated the role of miR-133a-1 in regulating NLRP3 inflammasome activation. miR-133a-1 indirectly controlled activation of the NLRP3 inflammasome via the suppression of mitochondrial uncoupling protein 2 (UCP2) without modifying the basal expression of the inflammasome components NLRP3 and ASC, the effector caspase-1, and the downstream IL-1β cytokine [179].

NLRP3-NEK7 interaction is required in NLRP3 oligomerization and activation downstream of the K^+^ efflux, and is induced by several stimuli. In the study conducted by Wu and colleagues, they reported that the overexpression of miR-200c alleviated cell inflammation and pyroptosis related to the NLRP3 inflammasome and ameliorated DSS-induced murine colitis symptoms via NEK7 modulation [180].

Decreased expression levels of miR-378a-5p in UC patients, and its potential involvement in IBD pathology, were reported [187]. The increment of miRNA-378a-5p, carried by MSC-EVs, suppressed the activation of the NLRP3 inflammasome, resulting in the inhibition of cellular pyroptosis [184].

miR-22 is downregulated in CRC tissues; it is involved in proliferation, colony formation, and the migration and invasion of CRC cells [188]. The overexpression of miR-22 decreased the expression of NLRP3, suppressing cell proliferation, migration, and invasion. Moreover, in a CRC mice model, miR-22 retards tumor growth and reduces the expression of Ki-67 in tumor tissue by targeting NLRP3 [181].

The ubiquitin system regulates the activation of the NLRP3 inflammasome. In this context, the enzyme BRCC3 promotes the activation of the NLRP3 inflammasome through its deubiquitination [26]. It was demonstrated that miR-369-3p modulated the activation of NLRP3 inflammasomes through the inhibition of BRCC3 expression. As a result, miR-369-3p decreased the recruitment of the ASC protein, the activity of Caspase-1, and the release of IL-1β and IL-18 [26].

## 5. Conclusions

Inflammation is the defensive response by the immune system to attenuate tissue damage or infections of critical importance to maintaining intestinal homeostasis. Inflammasomes and their signaling cascades play an essential role in inflammatory response and regulation of intestinal homeostasis; however, their aberrant activation may lead to the development of inflammatory disorders.

This review summarizes the structures, activation mechanisms, and impacts of inflammasome on various gastrointestinal diseases, as well as the potential therapeutic targets and strategies. Current research proves the active involvement of inflammasome in IBD pathogenesis, relating not only to the dysregulated activities of inflammasome, but also to the hereditary mutations in the gene-encoding sensors and effectors that increased the susceptibility to disease. Investigation on CRC allows us to understand the inflammasomes involvement in tumor induction and progression by creating a favorable TME and EMT-promoting process. Further evidence shows the involvement of inflammasomes also in the immunopathogenesis of IBS and celiac disease, even though insights in this field are still limited. Thus, targeting upstream and downstream inflammasome pathways in the gut appears to be a potential therapeutic approach in intestinal diseases.

Research on inflammasomes in gastrointestinal disease still has many unanswered questions. Inflammasome activation is a complex process that involves multiple signaling pathways. A full understanding of these mechanisms still poses a challenge. Furthermore, the mechanisms responsible for controlling inflammasomes activity and interactions with other immune pathways are not fully understood. To date, several pharmacological drugs, as well as natural compound inhibitors, have been documented. They ameliorate the inflammatory state, impair tissue damage, and increase therapeutic efficiency in combination with therapies in common use. However, inflammasome inhibitors are not yet applicable in clinical practice. There is a need for highly selective and potent inhibitors that target specific inflammasome components. In this context, miRNAs have the advantage of regulating inflammasomes that signal at multiple levels. A better understanding of their mechanisms of action could offer novel possible applications in the therapy of gastrointestinal diseases.

Currently, new opportunities for treating immune-mediated gastrointestinal inflammatory diseases could be developed to improve the options available in clinical practice.

## Figures and Tables

**Table 1:** Summary of the pharmacological inhibitors of inflammasomes and their ability to interfere with various steps of inflammasome activation in experimental models of intestinal disease.

Phase	Inhibitor	Target	Mechanism of Action	Experimental Models	References
Inflammasome Sensors	MCC950	NLRP3	Blocks canonical and non-canonical inflammasome activation binding NACHT domain and reduces IL-1β secretion	Spontaneous colitis mice	[101,102,103,104]
				DSS-induced colitis using Nod2 knockout mice	
				DSS-induced colitis mice	
	OLT117	NLRP3	Inhibits canonical and non-canonical inflammasome activation binding NACHT domain	DSS-induced colitis mice	[105]
	Tranilast	NLRP3	Influences the activation of inflammasome by binding NACHT domain Inhibits visceral hypersensitivity and colon permeability	DSS-induced colitis mice	[106,107,108,109]
				DSS-induced colitis rats	
				Mouse BMDMs and THP1	
				IBS rat models	
	G514-0208	NLRP3	Selectively inhibitor via NACHT domain binding	DSS-induced colitis mice	[110]
NF-κB Pathway	MCC950	NF-κB	In combination with metformin, inhibits the priming and activation of inflammasomes via TLR4/NF-κB and induces autophagy	DSS-induced colitis rats	[111]
	Dapagliflozin	NLRP3	Inhibits inflammasome activation via the NF-κB/AMPK/NLRP3 axis	Acetic acid-induced colitis in rats	[112]
	Canagliflozin	NLRP3	Inhibits inflammasome activation via the NF-κB/AMPK/NLRP3 axis	Acetic acid-induced colitis in rats.	[113]
	Oridonin	NF-κB	Inhibits inflammasome activation via NF-κB and reduced cytokines expression	TNBS-induced colitis mice	[114]
	Mirtazapine	NLRP3	Blocks inflammasome activation, modulating NF-κB activation and the NLRP3/caspase-1 signaling pathway	Acetic acid-induced colitis in rats	[115]
	BAY 11-7082	NLRP3	Inhibits inflammasome activation by suppressing both the NF-κB and NLRP3 pathways	Mouse BMDMs	[66,116]
				IBS rat models	
	Artemisinin	NLRP3	Blocks NLRP3 inflammasome assembly by inhibiting ROS production and NF-κB and MAPK signaling	Mouse BMDMs	[117]
				DSS-induced colitis mice	
Inflammasome Activation	Glyburide	K^+^ efflux	Prevents inflammasome activation through the inhibition of K^+^ efflux	Mouse BMDMs	[77,118]
				IL10^−/−^ mouse model	
	BHB	K^+^ efflux	Prevents inflammasome activation through the inhibition of K^+^ efflux and ASC oligomerization Reduces cytokine inflammasome-related release and mitigates pyroptosis	Mouse BMDMs	[119,120]
				DSS-induced colitis rats	
	(R,R)-BD-AcAc2	NLRP3	Prevents inflammasome activation and pyroptosis	DSS-induced colitis rats	[121]
	RRx-001	NEK7	Covalent binding to the cysteine residue, preventing NLRP3-NEK7 interaction; attenuates experimental colitis	Mouse BMDMs;	[122]
				DSS-induced colitis mice	
	WT161	NLRP3	Inhibits NLRP3 activation by blocking HDAC6	DSS-induced colitis mice	[123]
				Mouse BMDMs	
	C646	NLRP3	Inhibits NLRP3 activation by blocking histone acetyltransferase p300	DSS-induced colitis mice	[124]
				Mouse BMDMs	
	Metformin	NLRP3	Suppresses inflammatory processes and regulates the inflammatory microenvironment	LS1034	[125]
	BayK8644	TMEM176B	Triggers NLRP3 inflammasomes activation and limits the tumor growth	CRC mouse model	[126]
				THP-1	
				Mouse BMDMs	
Caspase-1 Activation and Pyroptosis	Artemisinin	NLRC4 AIM2	Inhibits Il-1β secretion	Mouse BMDMs	[117]
				DSS-induced colitis mice	
	Pralnacasan	Caspase-1	Inhibits caspase-1 activation	DSS-induced colitis mice	[30]
				Mouse BMDMs	
				THP-1	
	Ac-YVAD-cmk	Caspase-1	Inhibits caspase-1 activation	DSS-induced colitis mice	[127]
				Mouse BMDMs	
	Santacrumazate A	NLRP3	Inhibits HDAC2 expression and, in combination with antitumor agents, activates NLRP3/GSDMD-mediated pyroptosis	SW620	[128]
				LS174T	
	Lobaplatin	GSDME	Induces pyroptosis via GSDME activation	HT29	[129]
				HCT116	
	Simvastatin	NLRP3	Induces intracellular ROS production and triggers the NLRP3/caspase-1 pathway and pyroptosis	HCT116	[130]
				SW620	
	FL118	NLRP3	Induces NLRP3-ASC-caspase-1 mediated pyroptosis	SW480	[131]
				HT29	
	A438079	P2X7	Induces Bcl2/caspase-9/caspase-3 apoptosis and NLRP3/caspase-1 pyroptosis	HCT116	[132]
				SW620	

**Table 2:** Summary of natural compounds that modulate inflammasome assembly and activation in experimental intestinal disease.

Phase	Natural Compounds	Target	Mechanism of Action	Experimental Models	References
NF-κB Pathway	Naringin	NF-κB	Suppresses the activation of NF-κB, preventing the NLRP3 inflammasome signaling pathway	DSS-induced colitis mice	[140]
	Procyanidin	NF-κB	Suppresses the activation of NF-κB, preventing NLRP3 inflammasome activation	DSS-induced colitis mice	[141]
				THP-1	
	Phloretin	NF-κB	Suppresses the activation of NF-κB, preventing NLRP3 inflammasome activation	DSS-induced colitis mice	[142]
	Formononetin	NLRP3	Inhibits the NLRP3 signaling pathway	DSS-induced colitis mice	[143]
	Genistein	NLRP3	Inhibits the NLRP3 signaling pathway	DSS-induced colitis mice	[144]
				THP-1	
	Celastrol	NF-κB	Inhibits NLRP3 inflammasome activation and contrasts oxidative stress	DSS-induced colitis mice	[145]
	Evodiamine	NF-κB	Modulates NLRP3 inflammasome activation	DSS-induced colitis mice	[146]
	Bergenin	NF-κB	Blocks the canonical and non-canonical NLRP3 inflammasome pathways	DSS-induced colitis rats	[147]
	Secoisolariciresinol diglucoside	NF-κB	Inhibits NLRP1 inflammasome activation	DSS-induced colitis mice Raw264.7	[148]
Inflammasome Activation	Cardamonin	Nrf2	Inhibits NLRP3 inflammasome activation by inducing activation of the AhR/Nrf2/NQO1 pathway	DSS-induced colitis mice	[149]
				TNBS-induced colitis mice	
				BMDMs	
				THP-1	
	Dimethyl fumarate	Nrf2	Reduces NLRP3 inflammasome activation by inducing Nrf2 pathway activation	DSS-induced colitis mice	[150]
				THP-1	
	Coptisine	Nrf2	Modulates the expression levels of NLRP3 inflammasome components, increasing the expression of Nrf2	PI-IBS rats	[151]
	Luteolin	ROS	Inhibits NLRP3 inflammasomes activation and promotes macrophage polarization	Raw264.7	[152]
	Atractylenolide	DRP1	Inhibits NLRP3 inflammasome activation via mitochondrial damage suppression	AOM/DSS mice	[153]
	Parthenolide	NEK7 K^+^ efflux	Inhibits NLRP3 inflammasome assembly, affecting NLRP3/NEK7 interaction Inhibits NLRP3 inflammasome activation by suppressing K^+^ efflux	Murine BMDMs	[116,154]
				THP-1	
	Curcumin	K^+^ efflux	Inhibits activation of the NLRP3 inflammasome, reducing ROS and Cathepsin B release	DSS-induced colitis mice	[155]
	Vitamine D3	NEK7	Impairs NLRP3/NEK7 interaction, suppressing NLRP3 inflammasome assembly and activation	DSS-induced colitis mice	[156]
	Ginsenosides	NLRP3	Reduces NLRP3 inflammasome activation	DSS-induced colitis mice	[157,158]
	Salidroside	NLRP3	Modulates NLRP3 inflammasome activation and downstream pro-inflammatory mediators	DSS-induced colitis mice	[159]
	Palmatine	NLRP3	Modulates NLRP3 inflammasome activation and increases mitophagy	DSS-induced colitis mice	[160]
	Cinnamadehyde	NLRP3	Inhibits activation of the NLRP3 inflammasome and restores the NLRP3-related inflammatory mediators	DSS-induced colitis mice	[161,162]
	Paeoniflorin	miR-29a	Modulates NLRP3 inflammasome activation	IBS mice	[163]
	CJAF	NLRP3	Modulates NLRP3 inflammasome activation and downstream pro-inflammatory mediators	IBS mice	[164]
	Eugeniin	NLRC4	Reduces NAIP/NLRC4 inflammasome activation by bacterial pathogenic components	DSS-induced colitis mice	[38]
				Raw264.7	
				THP-1	
	Arctigenin	NLRP3	Inhibits NLRP3 inflammasome assembly and activation	AOM/DSS mice	[165]
	Andrographolide	NLRP3	Inhibits NLRP3 inflammasome activation In combination with antitumor agents, enhances the effects of cancer therapies	DSS-induced colitis mice	[166,167]
				AOM/DSS mice	
				Murine BMDMs	
	GL-V9	NLRP3	Triggers autophagy, inducing NLRP3 inflammasome degradation	DSS-induced colitis mice	[168]
				AOM/DSS mice	
				THP-1	
				Murine BMDMs	
Caspase-1 Activation and Pyroptosis	Luteolin	Caspase-1 GSDMD IL-1β	Inhibits cell proliferation and induces pyroptosis	HT29	[169]
	Curcumin	NLRP3	Promotes NLRP3-mediated pyroptosis	SW480	[170]
				HCT116	
				LoVo	
				HT29	
	Sulforaphane	Caspase-1	Inhibits caspase-1 cleavage and IL-1β maturation Reduces ROS levels	Raw264.7	[171,172]
				Murine BMDMs	
				DSS-induced colitis mice	
	VI-16	IL-1β	Inhibits NLRP3 inflammasome activation and IL-1β release	DSS-induced colitis mice	[173]
				THP-1	
Post-Transcriptional Modification	CAPE	NLRP3	Promotes NLRP3 ubiquitination and degradation	AOM/DSS mice	[165]

**Table 3:** Summary of miRNAs that interfere with various step of inflammasome in experimental intestinal disease.

Phase	miRNAs	Target	Mechanism of Action	Experimental Models	References
Inflammasome Activation	miR-223	NLRP3	Suppresses NLRP3 expression and activation	DSS-induced colitis mice	[178]
				Murine BMDMs	
				THP-1	
	miR-21 miR-155	NLRP3	Cinnamadehyde inhibits activation of the NLRP3 inflammasome by reducing the expression levels of miR-21 and miR-155	DSS-induced colitis mice	[161]
	miR-29a	NLRP3	Peoniflorin modulates NLRP3 inflammasome signaling by inhibiting miR-29a	IBS mice	[163]
	miR-133a-1	UCP2	Reduces the expression of NLRP3 inflammasome components and downstream cytokines	THP-1	[179]
	miR-200c-5p	NEK7	Reduces NLRP3 inflammasome activation and pyroptosis	DSS-induced colitis mice	[180]
	miR-22	NLRP3	Reduces NLRP3 inflammasome expression, cell proliferation, migration, and invasion	HCT116	[181]
				mouse xenograft model	
Pyroptosis	miR-223	NLRP3	Reduces IL-1β production and inhibits pyroptosis	DSS-induced colitis mice	[182,183]
				Murine BMDMs	
				THP-1	
	miR-378a-5p	NLRP3	Inhibits NLRP3 activation and pyroptosis	DSS-induced colitis mice	[184]
				Murine BMDMs	
				THP-1	
Post-Transcriptional Modification	miR-369-3p	BRCC3	Inhibits deubiquitination of NLRP3, modulating inflammasome assembly and activation	Murine BMDMs	[26]

## Data Availability

Not applicable.

## Supplementary Materials

The following supporting information can be downloaded at https://www.mdpi.com/article/10.3390/ijms252313058/s1.

## References

[1] Shao T., Hsu R., Rafizadeh D.L., Wang L., Bowlus C.L., Kumar N., Mishra J., Timilsina S., Ridgway W.M., Gershwin M.E. (2023). The gut ecosystem and immune tolerance. J. Autoimmun..

[2] Li D., Wu M. (2021). Pattern recognition receptors in health and diseases. Signal Transduct. Target. Ther..

[3] Dai Y., Zhou J., Shi C. (2023). Inflammasome: Structure, biological functions, and therapeutic targets. MedComm.

[4] Man S.M. (2018). Inflammasomes in the Gastrointestinal Tract: Infection, Cancer and Gut Microbiota Homeostasis. Nat. Rev. Gastroenterol. Hepatol..

[5] Martinon F., Burns K., Tschopp J. (2002). The Inflammasome. Mol. Cell.

[6] Zheng D., Liwinski T., Elinav E. (2020). Inflammasome Activation and Regulation: Toward a Better Understanding of Complex Mechanisms. Cell Discov..

[7] Rathinam V.A., Fitzgerald K.A. (2016). Inflammasome Complexes: Emerging Mechanisms and Effector Functions. Cell.

[8] Platnich J.M., Muruve D.A. (2019). NOD-like receptors and inflammasomes: A review of their canonical and non-canonical signaling pathways. Arch. Biochem. Biophys..

[9] Yu P., Zhang X., Liu N., Tang L., Peng C., Chen X. (2021). Pyroptosis: Mechanisms and Diseases. Signal Transduct. Target. Ther..

[10] Downs K.P., Nguyen H., Dorfleutner A., Stehlik C. (2020). An Overview of the Non-Canonical Inflammasome. Mol. Asp. Med..

[11] Babamale A.O., Chen S.-T. (2021). Nod-like Receptors: Critical Intracellular Sensors for Host Protection and Cell Death in Microbial and Parasitic Infections. Int. J. Mol. Sci..

[12] Broz P., Dixit V.M. (2016). Inflammasomes: Mechanism of Assembly, Regulation and Signalling. Nat. Rev. Immunol..

[13] Sundaram B., Tweedell R.E., Prasanth Kumar S., Kanneganti T.D. (2024). The NLR family of innate immune and cell death sensors. Immunity.

[14] Ohto U. (2022). Activation and regulation mechanisms of NOD-like receptors based on structural biology. Front. Immunol..

[15] Bauernfried S., Hornung V. (2022). Human NLRP1: From the shadows to center stage. J. Exp. Med..

[16] Sandstrom A., Mitchell P.S., Goers L., Mu E.W., Lesser C.F., Vance R.E. (2019). Functional Degradation: A Mechanism of NLRP1 Inflammasome Activation by Diverse Pathogen Enzymes. Science.

[17] Barry K., Murphy C., Mansell A. (2023). NLRP1—A Cinderella Story: A Perspective of Recent Advances in NLRP1 and the Questions They Raise. Commun. Biol..

[18] Burian M., Schmidt M.F., Yazdi A.S. (2023). The NLRP1 Inflammasome in Skin Diseases. Front. Immunol..

[19] Mi L., Min X., Chai Y., Zhang J., Chen X. (2022). NLRP1 Inflammasomes: A Potential Target for the Treatment of Several Types of Brain Injury. Front. Immunol..

[20] Williams T.M., Leeth R.A., Rothschild D.E., Coutermarsh-Ott S.L., McDaniel D.K., Simmons A.E., Heid B., Cecere T.E., Allen I.C. (2015). The NLRP1 Inflammasome Attenuates Colitis and Colitis-Associated Tumorigenesis. J. Immunol..

[21] Blevins H.M., Xu Y., Biby S., Zhang S. (2022). The NLRP3 Inflammasome Pathway: A Review of Mechanisms and Inhibitors for the Treatment of Inflammatory Diseases. Front. Aging Neurosci..

[22] Swanson K.V., Deng M., Ting J.P. (2019). The NLRP3 inflammasome: Molecular activation and regulation to therapeutics. Nat. Rev. Immunol..

[23] Kelley N., Jeltema D., Duan Y., He Y. (2019). The NLRP3 Inflammasome: An Overview of Mechanisms of Activation and Regulation. Int. J. Mol. Sci..

[24] Zhao N., Li C., Di B., Xu L. (2020). Recent Advances in the NEK7-Licensed NLRP3 Inflammasome Activation: Mechanisms, Role in Diseases and Related Inhibitors. J. Autoimmun..

[25] Xia J., Jiang S., Dong S., Liao Y., Zhou Y. (2023). The Role of Post-Translational Modifications in Regulation of NLRP3 Inflammasome Activation. Int. J. Mol. Sci..

[26] Scalavino V., Piccinno E., Valentini A.M., Schena N., Armentano R., Giannelli G., Serino G. (2023). miR-369-3p Modulates Intestinal Inflammatory Response via BRCC3/NLRP3 Inflammasome Axis. Cells.

[27] Li Z., Guo J., Bi L. (2020). Role of the NLRP3 Inflammasome in Autoimmune Diseases. Biomed. Pharmacother..

[28] Hamarsheh S., Zeiser R. (2020). NLRP3 Inflammasome Activation in Cancer: A Double-Edged Sword. Front. Immunol..

[29] Qiang R., Li Y., Dai X., Lv W. (2022). NLRP3 inflammasome in digestive diseases: From mechanism to therapy. Front. Immunol..

[30] Bauer C., Duewell P., Mayer C., Lehr H.A., Fitzgerald K.A., Dauer M., Tschopp J., Endres S., Latz E., Schnurr M. (2010). Colitis Induced in Mice with Dextran Sulfate Sodium (DSS) Is Mediated by the NLRP3 Inflammasome. Gut.

[31] Zhen Y., Zhang H. (2019). NLRP3 Inflammasome and Inflammatory Bowel Disease. Front. Immunol..

[32] Zaki M.H., Vogel P., Body-Malapel M., Lamkanfi M., Kanneganti T.-D. (2010). IL-18 Production Downstream of the Nlrp3 Inflammasome Confers Protection against Colorectal Tumor Formation. J. Immunol..

[33] Wang H., Wang Y., Du Q., Lu P., Fan H., Lu J., Hu R. (2016). Inflammasome-Independent NLRP3 Is Required for Epithelial-Mesenchymal Transition in Colon Cancer Cells. Exp. Cell Res..

[34] Poyet J.-L., Srinivasula S.M., Tnani M., Razmara M., Fernandes-Alnemri T., Alnemri E.S. (2001). Identification of Ipaf, a Human Caspase-1-Activating Protein Related to Apaf-1. J. Biol. Chem..

[35] Duncan J.A., Canna S.W. (2018). The NLRC4 Inflammasome. Immunol. Rev..

[36] Wen J., Xuan B., Liu Y., Wang L., He L., Meng X., Zhou T., Wang Y. (2021). Updating the NLRC4 Inflammasome: From Bacterial Infections to Autoimmunity and Cancer. Front. Immunol..

[37] Lei-Leston A.C., Murphy A.G., Maloy K.J. (2017). Epithelial Cell Inflammasomes in Intestinal Immunity and Inflammation. Front. Immunol..

[38] An Y., Zhai Z., Wang X., Ding Y., He L., Li L., Mo Q., Mu C., Xie R., Liu T. (2023). Targeting *Desulfovibrio vulgaris* Flagellin-Induced NAIP/NLRC4 Inflammasome Activation in Macrophages Attenuates Ulcerative Colitis. J. Adv. Res..

[39] Tong G., Shen Y., Li H., Qian H., Tan Z. (2024). NLRC4, inflammation and colorectal cancer (Review). Int. J. Oncol..

[40] Hu B., Elinav E., Huber S., Booth C.J., Strowig T., Jin C., Eisenbarth S.C., Flavell R.A. (2010). Inflammation-Induced Tumorigenesis in the Colon Is Regulated by Caspase-1 and NLRC4. Proc. Natl. Acad. Sci. USA.

[41] Liu R., Truax A.D., Chen L., Hu P., Li Z., Chen J., Song C., Chen L., Ting J.P.-Y. (2015). Expression Profile of Innate Immune Receptors, NLRs and AIM2, in Human Colorectal Cancer: Correlation with Cancer Stages and Inflammasome Components. Oncotarget.

[42] Allam R., Maillard M.H., Tardivel A., Chennupati V., Bega H., Yu C.W., Velin D., Schneider P., Maslowski K.M. (2015). Epithelial NAIPs Protect against Colonic Tumorigenesis. J. Exp. Med..

[43] Zheng D., Kern L., Elinav E. (2021). The NLRP6 inflammasome. Immunology.

[44] Chen G.Y., Liu M., Wang F., Bertin J., Núñez G. (2011). A Functional Role for Nlrp6 in Intestinal Inflammation and Tumorigenesis. J. Immunol..

[45] Levy M., Shapiro H., Thaiss C.A., Elinav E. (2017). NLRP6: A Multifaceted Innate Immune Sensor. Trends Immunol..

[46] Angosto-Bazarra D., Molina-López C., Pelegrín P. (2022). Physiological and pathophysiological functions of NLRP6: Pro- and anti-inflammatory roles. Commun. Biol..

[47] Wlodarska M., Thaiss C.A., Nowarski R., Henao-Mejia J., Zhang J.-P., Brown E.M., Frankel G., Levy M., Katz M.N., Philbrick W.M. (2014). NLRP6 Inflammasome Orchestrates the Colonic Host-Microbial Interface by Regulating Goblet Cell Mucus Secretion. Cell.

[48] Hu B., Elinav E., Huber S., Strowig T., Hao L., Hafemann A., Jin C., Wunderlich C., Wunderlich T., Eisenbarth S.C. (2013). Microbiota-Induced Activation of Epithelial IL-6 Signaling Links Inflammasome-Driven Inflammation with Transmissible Cancer. Proc. Natl. Acad. Sci. USA.

[49] Fan X., Jiao L., Jin T. (2022). Activation and Immune Regulation Mechanisms of PYHIN Family During Microbial Infection. Front. Microbiol..

[50] DeYoung K.L., Ray M.E., Su Y.A., Anzick S.L., Johnstone R.W., Trapani J.A., Meltzer P.S., Trent J.M. (1997). Cloning a Novel Member of the Human Interferon-Inducible Gene Family Associated with Control of Tumorigenicity in a Model of Human Melanoma. Oncogene.

[51] Lugrin J., Martinon F. (2018). The AIM 2 Inflammasome: Sensor of Pathogens and Cellular Perturbations. Immunol. Rev..

[52] Kumari P., Russo A.J., Shivcharan S., Rathinam V.A. (2020). AIM2 in health and disease: Inflammasome and beyond. Immunol. Rev..

[53] Jin T., Perry A., Jiang J., Smith P., Curry J.A., Unterholzner L., Jiang Z., Horvath G., Rathinam V.A., Johnstone R.W. (2012). Structures of the HIN Domain: DNA Complexes Reveal Ligand Binding and Activation Mechanisms of the AIM2 Inflammasome and IFI16 Receptor. Immunity.

[54] Lee S., Karki R., Wang Y., Nguyen L.N., Kalathur R.C., Kanneganti T.-D. (2021). AIM2 Forms a Complex with Pyrin and ZBP1 to Drive PANoptosis and Host Defence. Nature.

[55] Man S.M., Zhu Q., Zhu L., Liu Z., Karki R., Malik A., Sharma D., Li L., Malireddi R.K.S., Gurung P. (2015). Critical Role for the DNA Sensor AIM2 in Stem Cell Proliferation and Cancer. Cell.

[56] Chen J., Wang Z., Yu S. (2017). AIM2 Regulates Viability and Apoptosis in Human Colorectal Cancer Cells via the PI3K/Akt Pathway. OncoTargets Ther..

[57] Zhang Z., Li X., Zhang Y., Zhu H., Qiao Z., Lu Y., Mi X., Cao H., Shen G., He S. (2024). Absent in Melanoma 2 Attenuates Proliferation and Migration and Promotes Apoptosis of Human Colorectal Cancer Cells by Activating P38MAPK Signaling Pathway. Oncol. Res..

[58] Monroe K.M., Yang Z., Johnson J.R., Geng X., Doitsh G., Krogan N.J., Greene W.C. (2014). IFI16 DNA Sensor Is Required for Death of Lymphoid CD4 T Cells Abortively Infected with HIV. Science.

[59] Heilig R., Broz P. (2018). Function and Mechanism of the Pyrin Inflammasome. Eur. J. Immunol..

[60] Papadopoulos V.P., Antoniadou C., Ritis K., Skendros P. (2022). MEFV Mutations in IBD Patients: A Systematic Review and Meta- analysis. J. Gastrointest. Liver Dis..

[61] Sharma B.R., Kanneganti T.D. (2023). Inflammasome signaling in colorectal cancer. Transl. Res..

[62] Camilleri M. (2021). Diagnosis and Treatment of Irritable Bowel Syndrome: A Review. JAMA.

[63] Shaikh S.D., Sun N., Canakis A., Park W.Y., Weber H.C. (2023). Irritable Bowel Syndrome and the Gut Microbiome: A Comprehensive Review. J. Clin. Med..

[64] Berumen A., Edwinson A.L., Grover M. (2021). Post-infection Irritable Bowel Syndrome. Gastroenterol. Clin. N. Am..

[65] Gu Q.-Y., Zhang J., Feng Y.-C. (2016). Role of NLRP3 Inflammasome in *Bifidobacterium longum*-Regulated Visceral Hypersensitivity of Postinfectious Irritable Bowel Syndrome. Artif. Cells Nanomed. Biotechnol..

[66] Scuderi S.A., Casili G., Lanza M., Filippone A., Paterniti I., Esposito E., Campolo M. (2020). Modulation of NLRP3 Inflammasome Attenuated Inflammatory Response Associated to Diarrhea-Predominant Irritable Bowel Syndrome. Biomedicines.

[67] Yu L.M., Zhao K.J., Wang S.S., Wang X., Lu B. (2019). Corticotropin-releasing Factor Induces Inflammatory Cytokines via the NLRP6-inflammatory Cytokine Axis in a Murine Model of Irritable Bowel Syndrome. J. Dig. Dis..

[68] Sun Y., Zhang M., Chen C., Gillilland M., Sun X., El–Zaatari M., Huffnagle G.B., Young V.B., Zhang J., Hong S. (2013). Stress-Induced Corticotropin-Releasing Hormone-Mediated NLRP6 Inflammasome Inhibition and Transmissible Enteritis in Mice. Gastroenterology.

[69] Caio G., Volta U., Sapone A., Leffler D.A., De Giorgio R., Catassi C., Fasano A. (2019). Celiac Disease: A Comprehensive Current Review. BMC Med..

[70] Iversen R., Sollid L.M. (2023). The Immunobiology and Pathogenesis of Celiac Disease. Annu. Rev. Pathol. Mech. Dis..

[71] Garrote J.A., Gómez-González E., Bernardo D., Arranz E., Chirdo F. (2008). Celiac Disease Pathogenesis: The Proinflammatory Cytokine Network. J. Pediatr. Gastroenterol. Nutr..

[72] Palová-Jelínková L., Dáňová K., Drašarová H., Dvořák M., Funda D.P., Fundová P., Kotrbová-Kozak A., Černá M., Kamanová J., Martin S.F. (2013). Pepsin Digest of Wheat Gliadin Fraction Increases Production of IL-1β via TLR4/MyD88/TRIF/MAPK/NF-ΚB Signaling Pathway and an NLRP3 Inflammasome Activation. PLoS ONE.

[73] Gómez Castro M.F., Miculán E., Herrera M.G., Ruera C., Perez F., Prieto E.D., Barrera E., Pantano S., Carasi P., Chirdo F.G. (2019). P31-43 Gliadin Peptide Forms Oligomers and Induces NLRP3 Inflammasome/Caspase 1-Dependent Mucosal Damage in Small Intestine. Front. Immunol..

[74] Ruera C.N., Perez F., Iribarren M.L., Guzman L., Menendez L., Garbi L., Chirdo F.G. (2023). Coexistence of Apoptosis, Pyroptosis, and Necroptosis Pathways in Celiac Disease. Clin. Exp. Immunol..

[75] Pontillo A., Vendramin A., Catamo E., Fabris A., Crovella S. (2011). The Missense Variation Q705K in CIAS1/NALP3/NLRP3 Gene and an NLRP1 Haplotype Are Associated with Celiac Disease. Am. J. Gastroenterol..

[76] Diez-Martin E., Hernandez-Suarez L., Muñoz-Villafranca C., Martin-Souto L., Astigarraga E., Ramirez-Garcia A., Barreda-Gómez G. (2024). Inflammatory Bowel Disease: A Comprehensive Analysis of Molecular Bases, Predictive Biomarkers, Diagnostic Methods, and Therapeutic Options. Int. J. Mol. Sci..

[77] Liu L., Dong Y., Ye M., Jin S., Yang J., Joosse M.E., Sun Y., Zhang J., Lazarev M., Brant S.R. (2016). The Pathogenic Role of NLRP3 Inflammasome Activation in Inflammatory Bowel Diseases of Both Mice and Humans. J. Crohn’s Colitis.

[78] Tourkochristou E., Aggeletopoulou I., Konstantakis C., Triantos C. (2019). Role of NLRP3 inflammasome in inflammatory bowel diseases. World J. Gastroenterol..

[79] Shao B.Z., Wang S.L., Pan P., Yao J., Wu K., Li Z.S., Bai Y., Linghu E.Q. (2019). Targeting NLRP3 Inflammasome in Inflammatory Bowel Disease: Putting out the Fire of Inflammation. Inflammation.

[80] Gao S.-J. (2015). Interleukin-18 Genetic Polymorphisms Contribute Differentially to the Susceptibility to Crohn’s Disease. World J. Gastroenterol..

[81] Siegmund B., Lehr H.-A., Fantuzzi G., Dinarello C.A. (2001). IL-1β-Converting Enzyme (Caspase-1) in Intestinal Inflammation. Proc. Natl. Acad. Sci. USA.

[82] Ishikura T., Kanai T., Uraushihara K., Iiyama R., Makita S., Totsuka T., Yamazaki M., Sawada T., Nakamura T., Miyata T. (2003). Interleukin-18 Overproduction Exacerbates the Development of Colitis with Markedly Infiltrated Macrophages in Interleukin-18 Transgenic Mice. J. Gastroenterol. Hepatol..

[83] Steiner A., Reygaerts T., Pontillo A., Ceccherini I., Moecking J., Moghaddas F., Davidson S., Caroli F., Grossi A., Castro F.F.M. (2022). Recessive NLRC4-Autoinflammatory Disease Reveals an Ulcerative Colitis Locus. J. Clin. Immunol..

[84] Ringel-Scaia V.M., Qin Y., Thomas C.A., Huie K.E., McDaniel D.K., Eden K., Wade P.A., Allen I.C. (2019). Maternal Influence and Murine Housing Confound Impact of NLRP1 Inflammasome on Microbiome Composition. J. Innate Immun..

[85] Cummings J.R.F., Cooney R.M., Clarke G., Beckly J., Geremia A., Pathan S., Hancock L., Guo C., Cardon L.R., Jewell D.P. (2010). The Genetics of NOD-like Receptors in Crohn’s Disease. Tissue Antigens.

[86] Chang L., Tian Y., Xu L., Hao Q., Song L., Lu Y., Zhen Y. (2023). Spotlight on NLRP6 and Tumor Research Situation: A Potential Cancer Participant. J. Immunol. Res..

[87] Lemire P., Robertson S.J., Maughan H., Tattoli I., Streutker C.J., Platnich J.M., Muruve D.A., Philpott D.J., Girardin S.E. (2017). The NLR Protein NLRP6 Does Not Impact Gut Microbiota Composition. Cell Rep..

[88] Mamantopoulos M., Ronchi F., Van Hauwermeiren F., Vieira-Silva S., Yilmaz B., Martens L., Saeys Y., Drexler S.K., Yazdi A.S., Raes J. (2017). Nlrp6- and ASC-Dependent Inflammasomes Do Not Shape the Commensal Gut Microbiota Composition. Immunity.

[89] Hu S., Peng L., Kwak Y.-T., Tekippe E.M., Pasare C., Malter J.S., Hooper L.V., Zaki H. (2015). The DNA Sensor AIM2 Maintains Intestinal Homeostasis via Regulation of Epithelial Antimicrobial Host Defense. Cell Rep..

[90] Ratsimandresy R.A., Indramohan M., Dorfleutner A., Stehlik C. (2017). The AIM2 Inflammasome Is a Central Regulator of Intestinal Homeostasis through the IL-18/IL-22/STAT3 Pathway. Cell. Mol. Immunol..

[91] Postwala H., Shah Y., Parekh P.S., Chorawala M.R. (2023). Unveiling the genetic and epigenetic landscape of colorectal cancer: New insights into pathogenic pathways. Med. Oncol..

[92] Bhat A.A., Nisar S., Singh M., Ashraf B., Masoodi T., Prasad C.P., Sharma A., Maacha S., Karedath T., Hashem S. (2022). Cytokine- and Chemokine-induced Inflammatory Colorectal Tumor Microenvironment: Emerging Avenue for Targeted Therapy. Cancer Commun..

[93] Shao X., Lei Z., Zhou C. (2020). NLRP3 Promotes Colorectal Cancer Cell Proliferation and Metastasis via Regulating Epithelial Mesenchymal Transformation. Anticancer Agents Med. Chem..

[94] Hong S., Hwang I., Lee Y.-S., Park S., Lee W.-K., Fernandes-Alnemri T., Alnemri E.S., Kim Y.-S., Yu J.-W. (2013). Restoration of ASC Expression Sensitizes Colorectal Cancer Cells to Genotoxic Stress-Induced Caspase-Independent Cell Death. Cancer Lett..

[95] Salcedo R., Worschech A., Cardone M., Jones Y., Gyulai Z., Dai R.-M., Wang E., Ma W., Haines D., O’hUigin C. (2010). MyD88-Mediated Signaling Prevents Development of Adenocarcinomas of the Colon: Role of Interleukin 18. J. Exp. Med..

[96] Sharma D., Malik A., Guy C.S., Karki R., Vogel P., Kanneganti T.-D. (2018). Pyrin Inflammasome Regulates Tight Junction Integrity to Restrict Colitis and Tumorigenesis. Gastroenterology.

[97] Dihlmann S., Tao S., Echterdiek F., Herpel E., Jansen L., Chang-Claude J., Brenner H., Hoffmeister M., Kloor M. (2014). Lack of Absent in Melanoma 2 (AIM2) Expression in Tumor Cells Is Closely Associated with Poor Survival in Colorectal Cancer Patients. Int. J. Cancer.

[98] Patsos G., Germann A., Gebert J., Dihlmann S. (2010). Restoration of Absent in Melanoma 2 (AIM2) Induces G2/M Cell Cycle Arrest and Promotes Invasion of Colorectal Cancer Cells. Int. J. Cancer.

[99] Yang Y., Zhang M., Jin C., Ding Y., Yang M., Wang R., Zhou Y., Zhou Y., Li T., Wang K. (2019). Absent in Melanoma 2 Suppresses Epithelial-mesenchymal Transition via Akt and Inflammasome Pathways in Human Colorectal Cancer Cells. J. Cell. Biochem..

[100] Wilson J.E., Petrucelli A.S., Chen L., Koblansky A.A., Truax A.D., Oyama Y., Rogers A.B., Brickey W.J., Wang Y., Schneider M. (2015). Inflammasome-Independent Role of AIM2 in Suppressing Colon Tumorigenesis via DNA-PK and Akt. Nat. Med..

[101] Coll R.C., Hill J.R., Day C.J., Zamoshnikova A., Boucher D., Massey N.L., Chitty J.L., Fraser J.A., Jennings M.P., Robertson A.A.B. (2019). MCC950 Directly Targets the NLRP3 ATP-Hydrolysis Motif for Inflammasome Inhibition. Nat. Chem. Biol..

[102] Perera A.P., Fernando R., Shinde T., Gundamaraju R., Southam B., Sohal S.S., Robertson A.A.B., Schroder K., Kunde D., Eri R. (2018). MCC950, a Specific Small Molecule Inhibitor of NLRP3 Inflammasome Attenuates Colonic Inflammation in Spontaneous Colitis Mice. Sci. Rep..

[103] Umiker B., Lee H.-H., Cope J., Ajami N.J., Laine J.-P., Fregeau C., Ferguson H., Alves S.E., Sciammetta N., Kleinschek M. (2019). The NLRP3 Inflammasome Mediates DSS-Induced Intestinal Inflammation in *Nod2* Knockout Mice. Innate Immun..

[104] Mehto S., Jena K.K., Nath P., Chauhan S., Kolapalli S.P., Das S.K., Sahoo P.K., Jain A., Taylor G.A., Chauhan S. (2019). The Crohn’s Disease Risk Factor IRGM Limits NLRP3 Inflammasome Activation by Impeding Its Assembly and by Mediating Its Selective Autophagy. Mol. Cell.

[105] Oizumi T., Mayanagi T., Toya Y., Sugai T., Matsumoto T., Sobue K. (2022). NLRP3 Inflammasome Inhibitor OLT1177 Suppresses Onset of Inflammation in Mice with Dextran Sulfate Sodium-Induced Colitis. Dig. Dis. Sci..

[106] Huang Y., Jiang H., Chen Y., Wang X., Yang Y., Tao J., Deng X., Liang G., Zhang H., Jiang W. (2018). Tranilast Directly Targets NLRP 3 to Treat Inflammasome-driven Diseases. EMBO Mol. Med..

[107] Seto Y., Kato K., Tsukada R., Suzuki H., Kaneko Y., Kojo Y., Sato H., Onoue S. (2017). Protective Effects of Tranilast on Experimental Colitis in Rats. Biomed. Pharmacother..

[108] Sun X., Suzuki K., Nagata M., Kawauchi Y., Yano M., Ohkoshi S., Matsuda Y., Kawachi H., Watanabe K., Asakura H. (2010). Rectal Administration of Tranilast Ameliorated Acute Colitis in Mice through Increased Expression of Heme Oxygenase-1. Pathol. Int..

[109] Nozu T., Arie H., Miyagishi S., Ishioh M., Takakusaki K., Okumura T. (2024). Tranilast Alleviates Visceral Hypersensitivity and Colonic Hyperpermeability by Suppressing NLRP3 Inflammasome Activation in Irritable Bowel Syndrome Rat Models. Int. Immunopharmacol..

[110] Dai Z., Chen X., An L., Li C., Zhao N., Yang F., You S., Hou C., Li K., Jiang C. (2021). Development of Novel Tetrahydroquinoline Inhibitors of NLRP3 Inflammasome for Potential Treatment of DSS-Induced Mouse Colitis. J. Med. Chem..

[111] Saber S., El-Kader E.M.A. (2021). Novel Complementary Coloprotective Effects of Metformin and MCC950 by Modulating HSP90/NLRP3 Interaction and Inducing Autophagy in Rats. Inflammopharmacology.

[112] El-Rous M.A., Saber S., Raafat E.M., Ahmed A.A.E. (2021). Dapagliflozin, an SGLT2 Inhibitor, Ameliorates Acetic Acid-Induced Colitis in Rats by Targeting NFκB/AMPK/NLRP3 Axis. Inflammopharmacology.

[113] Nasr M., Cavalu S., Saber S., Youssef M.E., Abdelhamid A.M., Elagamy H.I., Kamal I., Gaafar A.G.A., El-Ahwany E., Amin N.A. (2022). Canagliflozin-Loaded Chitosan-Hyaluronic Acid Microspheres Modulate AMPK/NF-ΚB/NLRP3 Axis: A New Paradigm in the Rectal Therapy of Ulcerative Colitis. Biomed. Pharmacother..

[114] Liu Q.Q., Wang H.L., Chen K., Wang S.B., Xu Y., Ye Q., Sun Y.W. (2016). Oridonin Derivative Ameliorates Experimental Colitis by Inhibiting Activated T-cells and Translocation of Nuclear Factor-kappa B. J. Dig. Dis..

[115] Hafez H.M., Ibrahim M.A., Yehia Abdelzaher W., Gad A.A., Mohammed Naguib Abdel Hafez S., Abdel-Gaber S.A. (2021). Protective Effect of Mirtazapine against Acetic Acid-Induced Ulcerative Colitis in Rats: Role of NLRP3 Inflammasome Pathway. Int. Immunopharmacol..

[116] Juliana C., Fernandes-Alnemri T., Wu J., Datta P., Solorzano L., Yu J.-W., Meng R., Quong A.A., Latz E., Scott C.P. (2010). Anti-Inflammatory Compounds Parthenolide and Bay 11-7082 Are Direct Inhibitors of the Inflammasome. J. Biol. Chem..

[117] Hua L., Liang S., Zhou Y., Wu X., Cai H., Liu Z., Ou Y., Chen Y., Chen X., Yan Y. (2022). Artemisinin-Derived Artemisitene Blocks ROS-Mediated NLRP3 Inflammasome and Alleviates Ulcerative Colitis. Int. Immunopharmacol..

[118] Lamkanfi M., Mueller J.L., Vitari A.C., Misaghi S., Fedorova A., Deshayes K., Lee W.P., Hoffman H.M., Dixit V.M. (2009). Glyburide Inhibits the Cryopyrin/Nalp3 Inflammasome. J. Cell Biol..

[119] Youm Y.-H., Nguyen K.Y., Grant R.W., Goldberg E.L., Bodogai M., Kim D., D’Agostino D., Planavsky N., Lupfer C., Kanneganti T.D. (2015). The Ketone Metabolite β-Hydroxybutyrate Blocks NLRP3 Inflammasome–Mediated Inflammatory Disease. Nat. Med..

[120] Abdelhady R., Saber S., Ahmed Abdel-Reheim M., Alamri M.M.S., Alfaifi J., Adam M.I.E., Saleh L.A., Farag A.I., Elmorsy E.A., El-Wakeel H.S. (2023). Unveiling the Therapeutic Potential of Exogenous β-Hydroxybutyrate for Chronic Colitis in Rats: Novel Insights on Autophagy, Apoptosis, and Pyroptosis. Front. Pharmacol..

[121] Saber S., Alamri M.M.S., Alfaifi J., Saleh L.A., Abdel-Ghany S., Aboregela A.M., Farrag A.A., Almaeen A.H., Adam M.I.E., AlQahtani A.A.J. (2023). (R,R)-BD-AcAc2 Mitigates Chronic Colitis in Rats: A Promising Multi-Pronged Approach Modulating Inflammasome Activity, Autophagy, and Pyroptosis. Pharmaceuticals.

[122] Chen Y., He H., Lin B., Chen Y., Deng X., Jiang W., Zhou R. (2021). RRx-001 Ameliorates Inflammatory Diseases by Acting as a Potent Covalent NLRP3 Inhibitor. Cell. Mol. Immunol..

[123] Long X., Yu X., Gong P., Wang X., Tian L. (2022). Identification of WT161 as a Potent Agent for the Treatment of Colitis by Targeting the Nucleotide-Binding Domain-Like Receptor Family Pyrin Domain Containing 3 Inflammasome. Front. Pharmacol..

[124] Xu X., Li J., Long X., Tao S., Yu X., Ruan X., Zhao K., Tian L. (2021). C646 Protects Against DSS-Induced Colitis Model by Targeting NLRP3 Inflammasome. Front. Pharmacol..

[125] Liu G., Wang F., Feng Y., Tang H. (2024). Metformin Inhibits NLRP3 Inflammasome Expression and Regulates Inflammatory Microenvironment to Delay the Progression of Colorectal Cancer. Recent Pat. Anti-Cancer Drug Discov..

[126] Segovia M., Russo S., Jeldres M., Mahmoud Y.D., Perez V., Duhalde M., Charnet P., Rousset M., Victoria S., Veigas F. (2019). Targeting TMEM176B Enhances Antitumor Immunity and Augments the Efficacy of Immune Checkpoint Blockers by Unleashing Inflammasome Activation. Cancer Cell.

[127] Zhang J., Fu S., Sun S., Li Z., Guo B. (2014). Inflammasome Activation Has an Important Role in the Development of Spontaneous Colitis. Mucosal Immunol..

[128] Guan X., Liu R., Wang B., Xiong R., Cui L., Liao Y., Ruan Y., Fang L., Lu X., Yu X. (2024). Inhibition of HDAC2 Sensitises Antitumour Therapy by Promoting NLRP3/GSDMD-mediated Pyroptosis in Colorectal Cancer. Clin. Transl. Med..

[129] Yu J., Li S., Qi J., Chen Z., Wu Y., Guo J., Wang K., Sun X., Zheng J. (2019). Cleavage of GSDME by Caspase-3 Determines Lobaplatin-Induced Pyroptosis in Colon Cancer Cells. Cell Death Dis..

[130] Xie W., Peng M., Liu Y., Zhang B., Yi L., Long Y. (2023). Simvastatin Induces Pyroptosis via ROS/Caspase-1/GSDMD Pathway in Colon Cancer. Cell Commun. Signal..

[131] Tang Z., Ji L., Han M., Xie J., Zhong F., Zhang X., Su Q., Yang Z., Liu Z., Gao H. (2020). Pyroptosis Is Involved in the Inhibitory Effect of FL118 on Growth and Metastasis in Colorectal Cancer. Life Sci..

[132] Zhang Y., Li F., Wang L., Lou Y. (2021). A438079 Affects Colorectal Cancer Cell Proliferation, Migration, Apoptosis, and Pyroptosis by Inhibiting the P2X7 Receptor. Biochem. Biophys. Res. Commun..

[133] Hashemzehi M., Yavari N., Rahmani F., Asgharzadeh F., Soleimani A., Shakour N., Avan A., Hadizadeh F., Fakhraie M., Marjaneh R.M. (2021). Inhibition of Transforming Growth Factor-Beta by Tranilast Reduces Tumor Growth and Ameliorates Fibrosis in Colorectal Cancer. EXCLI J..

[134] Liang R., Chen W., Fan H., Chen X., Zhang J., Zhu J.-S. (2020). Dihydroartemisinin Prevents Dextran Sodium Sulphate-Induced Colitis through Inhibition of the Activation of NLRP3 Inflammasome and P38 MAPK Signaling. Int. Immunopharmacol..

[135] He Y., Zeng M.Y., Yang D., Motro B., Núñez G. (2016). NEK7 Is an Essential Mediator of NLRP3 Activation Downstream of Potassium Efflux. Nature.

[136] Magupalli V.G., Negro R., Tian Y., Hauenstein A.V., Di Caprio G., Skillern W., Deng Q., Orning P., Alam H.B., Maliga Z. (2020). HDAC6 Mediates an Aggresome-like Mechanism for NLRP3 and Pyrin Inflammasome Activation. Science.

[137] Fang Q., Xu Y., Tan X., Wu X., Li S., Yuan J., Chen X., Huang Q., Fu K., Xiao S. (2024). The Role and Therapeutic Potential of Pyroptosis in Colorectal Cancer: A Review. Biomolecules.

[138] Duarte J.A., de Barros A.L.B., Leite E.A. (2021). The Potential Use of Simvastatin for Cancer Treatment: A Review. Biomed. Pharmacother..

[139] Li M., Wang L., Wei Y., Wang W., Liu Z., Zuo A., Liu W., Tian J., Wang H. (2022). Anti-Colorectal Cancer Effects of a Novel Camptothecin Derivative PCC0208037 In Vitro and In Vivo. Pharmaceuticals.

[140] Cao H., Liu J., Shen P., Cai J., Han Y., Zhu K., Fu Y., Zhang N., Zhang Z., Cao Y. (2018). Protective Effect of Naringin on DSS-Induced Ulcerative Colitis in Mice. J. Agric. Food Chem..

[141] Chen L., You Q., Hu L., Gao J., Meng Q., Liu W., Wu X., Xu Q. (2018). The Antioxidant Procyanidin Reduces Reactive Oxygen Species Signaling in Macrophages and Ameliorates Experimental Colitis in Mice. Front. Immunol..

[142] Wu M., Li P., An Y., Ren J., Yan D., Cui J., Li D., Li M., Wang M., Zhong G. (2019). Phloretin Ameliorates Dextran Sulfate Sodium-Induced Ulcerative Colitis in Mice by Regulating the Gut Microbiota. Pharmacol. Res..

[143] Wu D., Wu K., Zhu Q., Xiao W., Shan Q., Yan Z., Wu J., Deng B., Xue Y., Gong W. (2018). Formononetin Administration Ameliorates Dextran Sulfate Sodium-Induced Acute Colitis by Inhibiting NLRP3 Inflammasome Signaling Pathway. Mediators Inflamm..

[144] Chen Y., Le T.H., Du Q., Zhao Z., Liu Y., Zou J., Hua W., Liu C., Zhu Y. (2019). Genistein Protects against DSS-Induced Colitis by Inhibiting NLRP3 Inflammasome via TGR5-CAMP Signaling. Int. Immunopharmacol..

[145] Shaker M.E., Ashamallah S.A., Houssen M.E. (2014). Celastrol Ameliorates Murine Colitis via Modulating Oxidative Stress, Inflammatory Cytokines and Intestinal Homeostasis. Chem. Biol. Interact..

[146] Shen P., Zhang Z., Zhu K., Cao H., Liu J., Lu X., Li Y., Jing Y., Yuan X., Fu Y. (2019). Evodiamine Prevents Dextran Sulfate Sodium-Induced Murine Experimental Colitis via the Regulation of NF-ΚB and NLRP3 Inflammasome. Biomed. Pharmacother..

[147] Lopes de Oliveira G.A., Alarcón de la Lastra C., Rosillo M.Á., Castejon Martinez M.L., Sánchez-Hidalgo M., Rolim Medeiros J.V., Villegas I. (2019). Preventive Effect of Bergenin against the Development of TNBS-Induced Acute Colitis in Rats Is Associated with Inflammatory Mediators Inhibition and NLRP3/ASC Inflammasome Signaling Pathways. Chem. Biol. Interact..

[148] Wang Z., Chen T., Yang C., Bao T., Yang X., He F., Zhang Y., Zhu L., Chen H., Rong S. (2020). Secoisolariciresinol Diglucoside Suppresses Dextran Sulfate Sodium Salt-Induced Colitis through Inhibiting NLRP1 Inflammasome. Int. Immunopharmacol..

[149] Wang K., Lv Q., Miao Y., Qiao S., Dai Y., Wei Z. (2018). Cardamonin, a Natural Flavone, Alleviates Inflammatory Bowel Disease by the Inhibition of NLRP3 Inflammasome Activation via an AhR/Nrf2/NQO1 Pathway. Biochem. Pharmacol..

[150] Liu X., Zhou W., Zhang X., Lu P., Du Q., Tao L., Ding Y., Wang Y., Hu R. (2016). Dimethyl Fumarate Ameliorates Dextran Sulfate Sodium-Induced Murine Experimental Colitis by Activating Nrf2 and Suppressing NLRP3 Inflammasome Activation. Biochem. Pharmacol..

[151] Xiong Y., Wei H., Chen C., Jiao L., Zhang J., Tan Y., Zeng L. (2022). Coptisine Attenuates Post-infectious IBS via Nrf2-dependent Inhibition of the NLPR3 Inflammasome. Mol. Med. Rep..

[152] Zhang B.-C., Li Z., Xu W., Xiang C.-H., Ma Y.-F. (2018). Luteolin Alleviates NLRP3 Inflammasome Activation and Directs Macrophage Polarization in Lipopolysaccharide-Stimulated RAW264.7 Cells. Am. J. Transl. Res..

[153] Qin Y., Yu Y., Yang C., Wang Z., Yang Y., Wang C., Zheng Q., Li D., Xu W. (2021). Atractylenolide I Inhibits NLRP3 Inflammasome Activation in Colitis-Associated Colorectal Cancer via Suppressing Drp1-Mediated Mitochondrial Fission. Front. Pharmacol..

[154] Liu L., Feng L., Gao J., Hu J., Li A., Zhu Y., Zhang C., Qiu B., Shen Z. (2023). Parthenolide Targets NLRP3 to Treat Inflammasome-Related Diseases. Int. Immunopharmacol..

[155] Gong Z., Zhao S., Zhou J., Yan J., Wang L., Du X., Li H., Chen Y., Cai W., Wu J. (2018). Curcumin Alleviates DSS-Induced Colitis via Inhibiting NLRP3 Inflammsome Activation and IL-1β Production. Mol. Immunol..

[156] Cao R., Ma Y., Li S., Shen D., Yang S., Wang X., Cao Y., Wang Z., Wei Y., Li S. (2020). 1,25(OH)_2_D_3_ Alleviates DSS-Induced Ulcerative Colitis via Inhibiting NLRP3 Inflammasome Activation. J. Leukoc. Biol..

[157] Liu C., Wang J., Yang Y., Liu X., Zhu Y., Zou J., Peng S., Le T.H., Chen Y., Zhao S. (2018). Ginsenoside Rd Ameliorates Colitis by Inducing P62-Driven Mitophagy-Mediated NLRP3 Inflammasome Inactivation in Mice. Biochem. Pharmacol..

[158] Tian M., Ma P., Zhang Y., Mi Y., Fan D. (2020). Ginsenoside Rk3 Alleviated DSS-Induced Ulcerative Colitis by Protecting Colon Barrier and Inhibiting NLRP3 Inflammasome Pathway. Int. Immunopharmacol..

[159] Liu J., Cai J., Fan P., Zhang N., Cao Y. (2019). The Abilities of Salidroside on Ameliorating Inflammation, Skewing the Imbalanced Nucleotide Oligomerization Domain-Like Receptor Family Pyrin Domain Containing 3/Autophagy, and Maintaining Intestinal Barrier Are Profitable in Colitis. Front. Pharmacol..

[160] Mai C.-T., Wu M.-M., Wang C.-L., Su Z.-R., Cheng Y.-Y., Zhang X.-J. (2019). Palmatine Attenuated Dextran Sulfate Sodium (DSS)-Induced Colitis via Promoting Mitophagy-Mediated NLRP3 Inflammasome Inactivation. Mol. Immunol..

[161] Qu S., Shen Y., Wang M., Wang X., Yang Y. (2019). Suppression of miR-21 and miR-155 of Macrophage by Cinnamaldehyde Ameliorates Ulcerative Colitis. Int. Immunopharmacol..

[162] Liu M.-L., Wong W.-T., Weng Y.-M., Ho C.-L., Hsu H.-T., Hua K.-F., Wu C.-H., Li L.-H. (2024). Cinnamaldehyde, a Bioactive Compound from the Leaves of *Cinnamomum osmophloeum* Kaneh, Ameliorates Dextran Sulfate Sodium-Induced Colitis in Mice by Inhibiting the NLRP3 Inflammasome. J. Physiol. Investig..

[163] Ke W., Wang Y., Huang S., Liu S., Zhu H., Xie X., Yang H., Lu Q., Gan J., He G. (2022). Paeoniflorin Alleviates Inflammatory Response in IBS-D Mouse Model via Downregulation of the NLRP3 Inflammasome Pathway with Involvement of miR-29a. Heliyon.

[164] Ke W., Wu J., Li H., Huang S., Li H., Wang Y., Wu Y., Yuan J., Zhang S., Tang H. (2024). Network Pharmacology and Experimental Validation to Explore the Mechanism of Changji’an Formula against Irritable Bowel Syndrome with Predominant Diarrhea. Heliyon.

[165] Dai G., Jiang Z., Sun B., Liu C., Meng Q., Ding K., Jing W., Ju W. (2020). Caffeic Acid Phenethyl Ester Prevents Colitis-Associated Cancer by Inhibiting NLRP3 Inflammasome. Front. Oncol..

[166] Guo W., Sun Y., Liu W., Wu X., Guo L., Cai P., Wu X., Wu X., Shen Y., Shu Y. (2014). Small Molecule-Driven Mitophagy-Mediated NLRP3 Inflammasome Inhibition Is Responsible for the Prevention of Colitis-Associated Cancer. Autophagy.

[167] Xu L., Cai P., Li X., Wu X., Gao J., Liu W., Yang J., Xu Q., Guo W., Gu Y. (2021). Inhibition of NLRP3 Inflammasome Activation in Myeloid-Derived Suppressor Cells by Andrographolide Sulfonate Contributes to 5-FU Sensitization in Mice. Toxicol. Appl. Pharmacol..

[168] Zhao Y., Guo Q., Zhao K., Zhou Y., Li W., Pan C., Qiang L., Li Z., Lu N. (2018). Small Molecule GL-V9 Protects against Colitis-Associated Colorectal Cancer by Limiting NLRP3 Inflammasome through Autophagy. Oncoimmunology.

[169] Chen Y., Ma S., Pi D., Wu Y., Zuo Q., Li C., Ouyang M. (2022). Luteolin Induces Pyroptosis in HT-29 Cells by Activating the Caspase1/Gasdermin D Signalling Pathway. Front. Pharmacol..

[170] Dal Z., Aru B. (2023). The Role of Curcumin on Apoptosis and NLRP3 Inflammasome-Dependent Pyroptosis on Colorectal Cancer in Vitro. Turk. J. Med. Sci..

[171] Greaney A.J., Maier N.K., Leppla S.H., Moayeri M. (2016). Sulforaphane Inhibits Multiple Inflammasomes through an Nrf2-Independent Mechanism. J. Leukoc. Biol..

[172] Zhou Z., Dong J., Qiu Y., Zhang G., Wei K., He L., Sun Y., Jiang H., Zhang S., Guo X. (2024). Sulforaphane Decreases Oxidative Stress and Inhibits NLRP3 Inflammasome Activation in a Mouse Model of Ulcerative Colitis. Biomed. Pharmacother..

[173] Zhao Y., Guo Q., Zhu Q., Tan R., Bai D., Bu X., Lin B., Zhao K., Pan C., Chen H. (2019). Flavonoid VI-16 Protects against DSS-Induced Colitis by Inhibiting Txnip-Dependent NLRP3 Inflammasome Activation in Macrophages via Reducing Oxidative Stress. Mucosal Immunol..

[174] Cascão R., Fonseca J.E., Moita L.F. (2017). Celastrol: A Spectrum of Treatment Opportunities in Chronic Diseases. Front. Med..

[175] Qiao S., Lv C., Tao Y., Miao Y., Zhu Y., Zhang W., Sun D., Yun X., Xia Y., Wei Z. (2020). Arctigenin Disrupts NLRP3 Inflammasome Assembly in Colonic Macrophages via Downregulating Fatty Acid Oxidation to Prevent Colitis-Associated Cancer. Cancer Lett..

[176] Deng Z., Rong Y., Teng Y., Mu J., Zhuang X., Tseng M., Samykutty A., Zhang L., Yan J., Miller D. (2017). Broccoli-Derived Nanoparticle Inhibits Mouse Colitis by Activating Dendritic Cell AMP-Activated Protein Kinase. Mol. Ther..

[177] O’Brien J., Hayder H., Zayed Y., Peng C. (2018). Overview of MicroRNA Biogenesis, Mechanisms of Actions, and Circulation. Front. Endocrinol..

[178] Bauernfeind F., Rieger A., Schildberg F.A., Knolle P.A., Schmid-Burgk J.L., Hornung V. (2012). NLRP3 Inflammasome Activity Is Negatively Controlled by miR-223. J. Immunol..

[179] Bandyopadhyay S., Lane T., Venugopal R., Parthasarathy P.T., Cho Y., Galam L., Lockey R., Kolliputi N. (2013). MicroRNA-133a-1 Regulates Inflammasome Activation through Uncoupling Protein-2. Biochem. Biophys. Res. Commun..

[180] Wu G., Zhang D., Yang L., Wu Q., Yuan L. (2022). MicroRNA-200c-5p Targets NIMA Related Kinase 7 (NEK7) to Inhibit NOD-like Receptor 3 (NLRP3) Inflammasome Activation, MODE-K Cell Pyroptosis, and Inflammatory Bowel Disease in Mice. Mol. Immunol..

[181] Cong J., Gong J., Yang C., Xia Z., Zhang H. (2020). miR-22 Suppresses Tumor Invasion and Metastasis in Colorectal Cancer by Targeting NLRP3. Cancer Manag. Res..

[182] Neudecker V., Haneklaus M., Jensen O., Khailova L., Masterson J.C., Tye H., Biette K., Jedlicka P., Brodsky K.S., Gerich M.E. (2017). Myeloid-Derived miR-223 Regulates Intestinal Inflammation via Repression of the NLRP3 Inflammasome. J. Exp. Med..

[183] Wu X., Pan S., Luo W., Shen Z., Meng X., Xiao M., Tan B., Nie K., Tong T., Wang X. (2020). Roseburiaintestinalis-derived Flagellin Ameliorates Colitis by Targeting miR-223-3p-mediated Activation of NLRP3 Inflammasome and Pyroptosis. Mol. Med. Rep..

[184] Cai X., Zhang Z., Yuan J., Ocansey D.K.W., Tu Q., Zhang X., Qian H., Xu W., Qiu W., Mao F. (2021). hucMSC-Derived Exosomes Attenuate Colitis by Regulating Macrophage Pyroptosis via the miR-378a-5p/NLRP3 Axis. Stem Cell Res. Ther..

[185] Polytarchou C., Oikonomopoulos A., Mahurkar S., Touroutoglou A., Koukos G., Hommes D.W., Iliopoulos D. (2015). Assessment of Circulating MicroRNAs for the Diagnosis and Disease Activity Evaluation in Patients with Ulcerative Colitis by Using the Nanostring Technology. Inflamm. Bowel Dis..

[186] Onisor D., Brusnic O., Banescu C., Carstea C., Sasaran M., Stoian M., Avram C., Boicean A., Boeriu A., Dobru D. (2024). miR-155 and miR-21 as Diagnostic and Therapeutic Biomarkers for Ulcerative Colitis: There Is Still a Long Way to Go. Biomedicines.

[187] Quaglio A.E.V., Santaella F.J., Rodrigues M.A.M., Sassaki L.Y., Di Stasi L.C. (2021). MicroRNAs Expression Influence in Ulcerative Colitis and Crohn’s Disease: A Pilot Study for the Identification of Diagnostic Biomarkers. World J. Gastroenterol..

[188] Xia S.-S., Zhang G.-J., Liu Z.-L., Tian H.-P., He Y., Meng C.-Y., Li L.-F., Wang Z.-W., Zhou T. (2017). MicroRNA-22 Suppresses the Growth, Migration and Invasion of Colorectal Cancer Cells through a Sp1 Negative Feedback Loop. Oncotarget.

