# Supplementary material for: Inflammasomes in Intestinal Disease: Mechanisms of Activation and Therapeutic Strategies
Source: Int J Mol Sci. 2024 Dec 4;25(23):13058. doi: 10.3390/ijms252313058 (PMC11642578; doi:10.3390/ijms252313058)

Figure S1. AIM2-like inflammasome activation. AIM2 recognizes and binds dsDNA through its HIN-200 domain, suppressing the AIM2 autoinhibitory state. The recruitment of the adapter protein ASC and effector pro-caspase-1 leads to inflammasome assembly. Consequently, the active form of caspase-1 induces IL-1ß and IL-18 activation and release and pyroptosis.

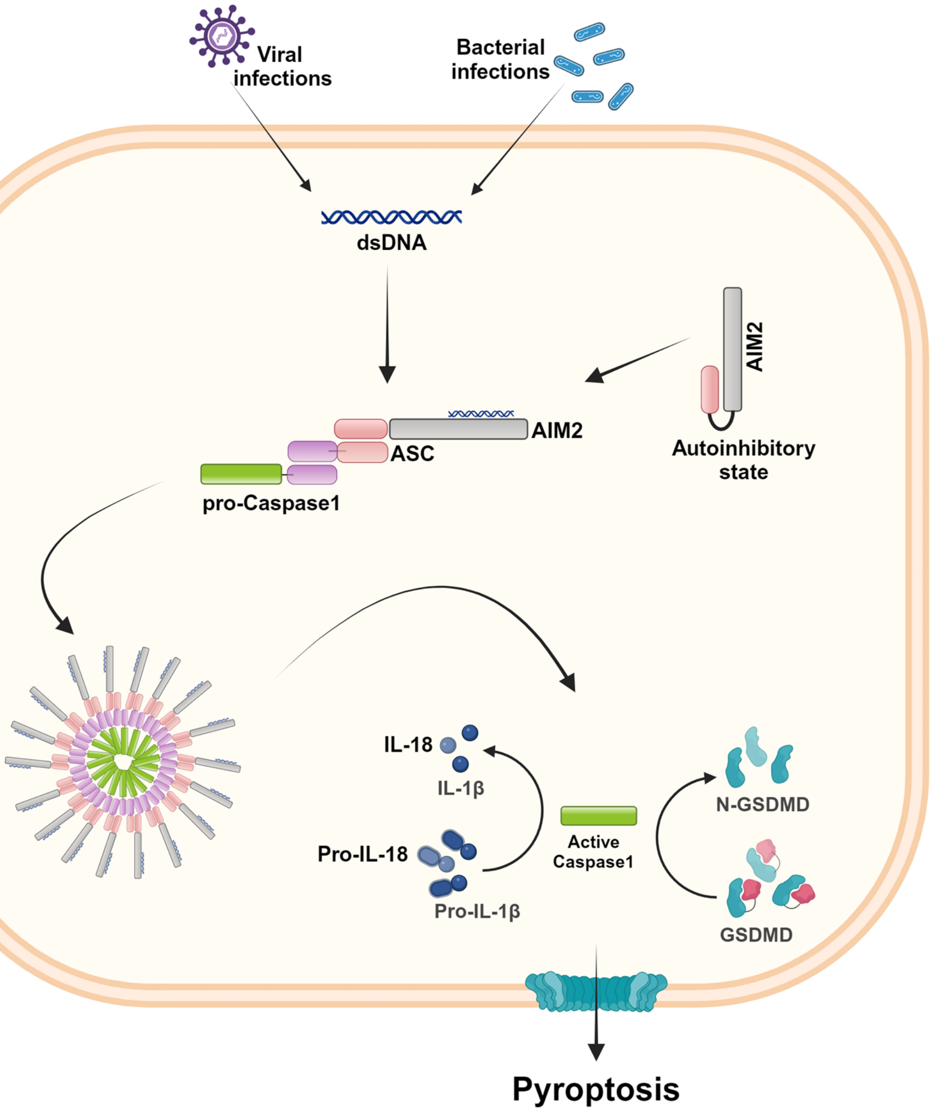

Figure S2. Pyrin inflammasomes activation. The activation of Pyrin is influenced by PAMPs, such as LPS, and cytokines (IFN-γ, TNF-α, IL-4 and IL-10) recognized through the C-terminal domain B30.2/SPRY. Upon activation Pyrin recruits the adapter protein ASC and the effector pro-caspase-1, leading to the activation and release of IL-1β and IL-18 and pyroptosis.

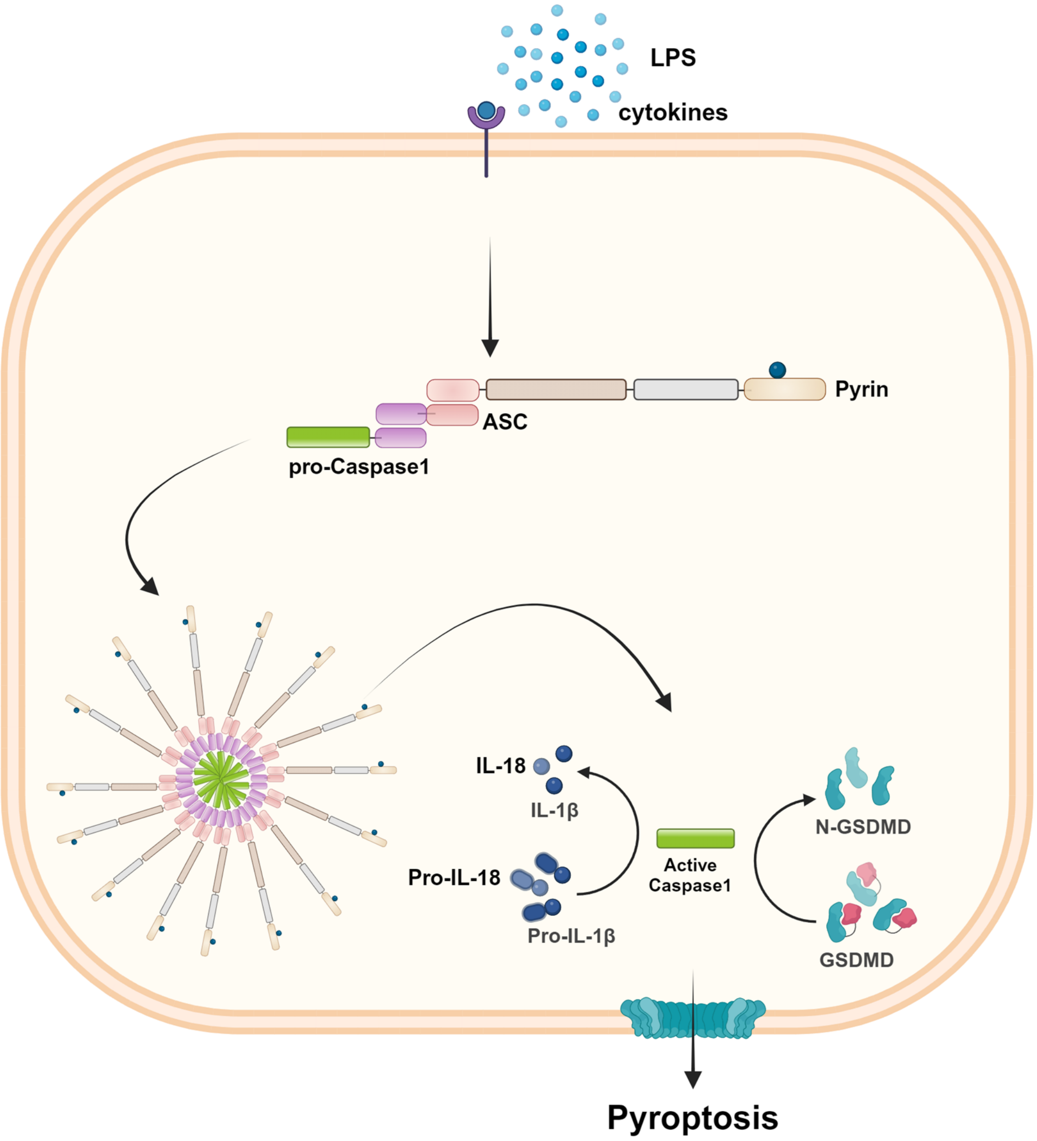

Supplement: Supplementary file 1 [file ijms-25-13058-s001.zip › ijms-3310195-supplementary.pdf]
